# Biologically Relevant Lighting: An Industry Perspective

**DOI:** 10.3389/fnins.2021.637221

**Published:** 2021-06-07

**Authors:** Robert Soler, Erica Voss

**Affiliations:** BIOS Lighting, Carlsbad, CA, United States

**Keywords:** social jet lag, circadian, lighting, melanopsin, Opn4

## Abstract

Innovations in LED lighting technology have led to tremendous adoption rates and vastly improved the metrics by which they are traditionally evaluated–including color quality, longevity, and energy efficiency to name a few. Additionally, scientific insight has broadened with respect to the biological impact of light, specifically our circadian rhythm. Indoor electric lighting, despite its many attributes, fails to specifically address the biological responses to light. Traditional electric lighting environments are biologically too dim during the day, too bright at night, and with many people spending much of their lives in these environments, it can lead to circadian dysfunction. The lighting industry’s biological solution has been to create bluer days and yellower nights, but the technology created to do so caters primarily to the cones. A better call to action is to provide biologically brighter days and biologically darker nights within the built environment. However, current lighting design practices have specified the comfort and utility of electric light. Brighter intensity during the day can often be uncomfortable or glary, and reduced light intensity at night may compromise visual comfort and safety, both of which will affect user compliance. No single lighting solution will effectively create biologically brighter days and biologically darker nights, but rather a variety of parameters need to be considered. This paper discusses the contributions of spectral power distribution, hue or color temperature, spatial distribution, as well as architectural geometry and surface reflectivity, to achieve biologically relevant lighting.

## Introduction

The discovery of a novel Intrinsically Photosenstive Retinal Ganglion Cell (ipRGC) in the eye has changed our understanding of the role of light in our everyday lives (Provencio and Foster, 1995; Provencio et al., 1998; Brainard et al., 2001; Thapan et al., 2001; Berson et al., 2002; Panda et al., 2002; Dacey et al., 2005; Güler et al., 2008; Hughes et al., 2016). These ipRGCs contain a photopigment melanopsin with an *in-vitro* spectral sensitivity around 480 nm. However, after lens transmission this spectral sensitivity is shifted to longer wavelengths between 487 and 496 nm (Spitschan, 2019), and it is now understood that these ipRGCs are responsible for several physiological effects such as circadian synchronization, tracking seasonal changes, acute alertness, working memory improvements and mood improvements (Gaggioni et al., 2014; LeGates et al., 2014; Rodgers et al., 2016; Fernandez et al., 2018; Brown, 2020). The role of rods and cones on ipRGC response is still being debated. Some studies have shown no contribution of S-cone (Spitschan et al., 2019) while others show an exposure time dependent contribution of S-cone (Brown et al., 2021) when evaluating melatonin suppression. Further studies show a reduced circadian impact of blue colored environments (Mouland et al., 2019). Less information is available on rod and cone contributions for other ipRGC driven responses.

In order to apply a biologically relevant lighting solution within the built environment, we must first understand the challenge at hand. Today, we spend most of our time indoors, removed from daylight which contains an important natural and robust daytime and nighttime signal (Knoop et al., 2020). Daylight has been replaced with electric lighting which has been engineered and optimized for visual efficiency and can be switched on or off at any time of the day. Moreover, today’s light level recommendations (while sufficient for visual tasks) are insufficient for proper circadian signaling. We find ourselves immersed in environments where the lighting is too biologically dim for our brains to receive a proper daytime signal and too biologically bright to provide a proper nighttime signal. This lack of delineation between day and night can lead to circadian drift and exacerbate social jet lag (Roenneberg and Merrow, 2016), which have been associated with a whole host of negative health outcomes: decreases in learning and attention, increased risk of obesity, addiction, and cardiovascular disease (Roenneberg et al., 2012; Zarrinpar et al., 2015; Roenneberg and Merrow, 2016; Sulli et al., 2018). Moreover, this appears to be a widespread phenomenon that affects the majority of people. In fact, it has been shown that 87% of non-shift workers have some form of circadian dysfunction and the associated health risks previously mentioned (Roenneberg and Merrow, 2016), illustrating that the current lighting environment may be insufficient for proper circadian entrainment and amplitude. Therefore, biologically relevant lighting should provide biologically brighter days and biologically darker nights within the built environment. However, in practice there are additional constraints. Energy consumption, visual comfort (i.e., glare) associated with brighter days, and visual acuity and safety related to darker nights. It should be noted that while everyone should benefit from brighter days and darker nights, it may not be additive, there may be more benefit from darker nights compared to brighter days (Skeldon et al., 2017), and vice versa, depending on existing conditions and population type. We do know that large differences exist between individuals for nighttime melatonin suppression (Phillips et al., 2019).

In order to create truly biologically relevant lighting, the following factors must be considered in conjunction with one another: spectral composition, color, intensity, and distribution of the light, as well as the geometry and reflectivity of the built environment. As long as we remain in the built environment, there is no single strategy that can provide the optimum circadian lighting environment. Instead, we must use a multi-faceted approach to achieve biologically relevant lighting that is focused on biologically brighter days and darker nights. Ultimately, we need to understand how both circadian lighting factors and visual lighting factors can be addressed to create spaces that are both biologically relevant for day and night while still providing comfortable and well-designed spaces (Berman et al., 1991; Rea and Ouellette, 1991).

## Current Industry Standards, Metrics and Recommendations

The WELL Building Standard^TM^ uses a series of design categories–Air, Water, Nourishment, Light, Movement, Thermal Comfort, Sound, Materials, Mind, Community, and Innovation–to create a point-based framework that determines how much wellness can be delivered to building occupants. A minimum of 40 points is required to achieve any type of WELL certification, with nine total points available from the Light concept. Circadian lighting and daylight exposure are key Features within the WELL^TM^ “Light” concept. The Circadian Lighting Design Feature uses vertical melanopic equivalent daylight illuminance (EDI) as a criterion for minimum daytime circadian stimulus. Vertical melanopic EDI is measured based on the occupants’ primary location within a space. Circadian light level measurements are meant to quantify the light reaching the occupants’ eye and as such are taken 4′ above finished floor (or 18″ above the task plane) in the primary viewing direction of the occupant. The amount of vertical melanopic EDI required is dependent on how much daylight availability there is. It ranges from 109 vertical melanopic EDI (for one point) when adequate daylight is present to 218 vertical melanopic EDI (for three points) when sufficient daylight is not present.

Melanopic EDI originated from The International Commission on Illumination (CIE) issuance of the CIE S 026/E:2018, and uses the same melanopic sensitivity function as the Lucas toolbox, with peak sensitivity at 490 nm. Additionally, the CIE has proposed melanopic Daylight Efficiency Ratio (melanopic DER) as a metric for determining the biological potential for a light spectrum to activate melanopsin relative to visual illuminance (lumens/m^2^) (Lucas et al., 2014; International Commission on Illumination, 2018).

## The Role of Led Technology in Biologically Relevant Lighting

Light emitting diodes (LEDs) lighting has seen tremendous adoption rates due to its energy efficiency and longevity. As of 2018 residential products saw LED penetration in 33% of A-lamps and 45% of recessed downlights, while office lighting saw LED penetration in 20% of linear installations (Navigant, 2019, 2020). How does LED lighting impact these newly discovered physiological responses to light? Some claim that LEDs contain a large blue peak, sending too many daytime signals and thus deeming them unsuitable for nighttime use (Tosini et al., 2016). Here I will demonstrate that the contrary is true and in fact, there exists a fundamental dip in the melanopic region (from 470 to 500 nm) due to the way LED white light spectrum is generated. This means that white LEDs actually perform poorly when it comes to producing the necessary melanopic stimulation relative to its perceived color temperature and visual brightness.

### Color Matching Functions

Color matching functions shown in Supplementary Figure 1 are used to convert any spectral power distribution to a (*x,y*) point on the CIE 1931 color space diagram. These (*x,y*) color points are how the lighting industry communicates color. When referring to white light, these color coordinates are binned based on the temperature of a black body radiator in Kelvin (referred to as K). The following equations are used to convert spectrum into Tristimulus *X*, *Y*, and *Z*, then to color coordinates (*x,y*). Tristimulus *Y* is also the luminous efficiency function, used to calculate lumens.

$$X=\sum_{\lambda =380\text{nm}}^{780\text{nm}}S\left(\lambda \right)\bar{x}\left(\lambda \right)\partial \lambda$$

$$Y=\sum_{\lambda =380\text{nm}}^{780\text{nm}}S\left(\lambda \right)\bar{y}\left(\lambda \right)\partial \lambda$$

$$Z=\sum_{\lambda =380\text{nm}}^{780\text{nm}}S\left(\lambda \right)\bar{z}\left(\lambda \right)\partial \lambda$$

Where

*S*(λ) = Spectral power distribution of the light source

**FIGURE 1:**
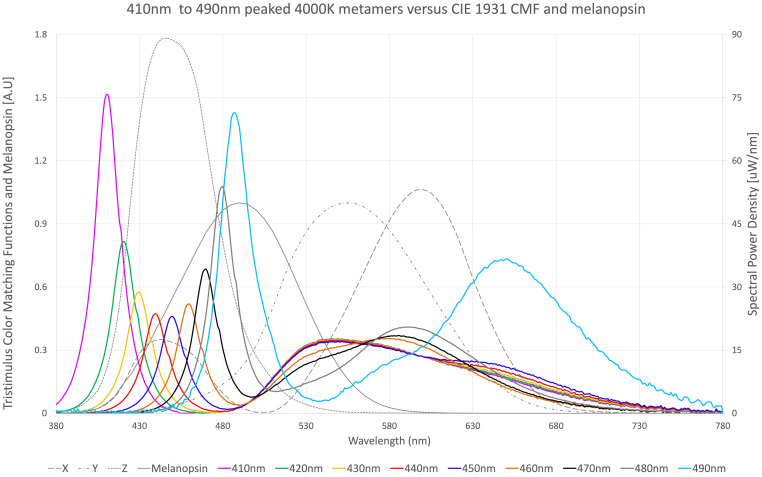
Nine spectral metamers of light, each with different blue light peaks. 410 nm peaked metamer (purple), 420 nm peaked metamer (green), 430 nm peaked metamer (yellow), 440 nm peaked metamer (red), 450 nm peaked metamer (royal blue), 460 nm peaked metamer (orange), 470 nm peaked metamer (black), 480 nm peaked metamer (gray thick), and 490 nm peaked metamer (sky blue) are overlayed on top of tristimulus color matching functions X (gray–long dash), Y (gray–dash dot), and Z (gray–small dash) according to CIE1931, as well as melanopsin sensitivity function (gray–solid) according to CIE S 026. Each metamer produces visually the same 4,000 K color of light at the same lumens according to the tristimulus functions.

$\bar{x}$(λ) = spectral tristimulus value from Figure 1.

$\bar{y}$(λ) = spectral tristimulus value from Figure 1.

$\bar{z}$(λ) = spectral tristimulus value from Figure 1.

$$x=\frac{X}{X+Y+Z}$$

$$y=\frac{\text{Y}}{X+Y+Z}$$

Where

*x* = *x*-coordinate on CIE 1931 color space diagram.

*y* = *y*-coordinate on CIE 1931 color space diagram.

One way to think about this is as follows: *Z* stimulation results in bluer color perception, *Y* stimulation results in greener color perception, and *X* stimulation results in redder color perception. This blue perception from *Z* stimulation versus melanopic stimulation is key to disentangling visual perception from physiological effect. *Z* has highest sensitivity from 430 to 450 nm, whereas melanopic blue-green has highest sensitivity from 480 to 500 nm. Thus, a spectral power distribution can be derived with heightened melanopic stimulation without appearing cold in color temperature. Likewise, a spectral power distribution can be created that has less melanopic stimulation while still appearing cold in color temperature.

### Traditional LED Technology

Light emitting diodes are semiconductors that utilize a material composition to emit a specific wavelength of light. There are two classes of LEDs; InGaN (blue) and AlInGaP (red), which are doped to produce a desired wavelength (Shaolin et al., 2015). However, a green gap exists in this doping process that makes green light very inefficient to produce from a discrete LED source (Auf Der Maur et al., 2016). Thus, a common approach to LED light is to utilize a monochromatic blue LED with peak emission between 445 and 455 nm combined with a combination of green and red phosphors that are excited by the blue wavelengths of the LED (Mueller-Mach et al., 2005; Nishiura et al., 2011; Liu et al., 2015). This combination is what provides the LED its significant energy efficiency and allows for a much wider range of CCTs and spectral control than previous technologies. An example of this approach is shown in Supplementary Figure 2 for a 6,500°K LED spectra. What can be seen from this LED spectra is a peak emission that coincides with the peak sensitivity for *Z* from the color matching functions. By pinpointing the *Z* sensitivity, LEDs provide the most visually blue-looking light source with the least amount of energy appropriated in the blue region of the spectrum, relative to other light sources. This is an important tactic for energy efficiency, as the Luminous Efficiency Function (tristimulus *y*) is very low in the blue region. A common misconception about LED lighting is that they produce more blue light than other sources, due to the narrow, high-amplitude peak of the blue wavelengths. However, this is not the case, LED light is in fact engineered to produce the least amount of blue light per lumen for any given color temperature of light, in order to gain the highest luminous efficacy.

### Spectral Simulation Method

Spectral simulation was created using an excel based spectrum database and calculator containing selectable Blue LED with scaler factor to increase or decrease LED simulated intensity. One Blue LED was selected and combined with various amounts of three unique phosphors. A combination of three (3) color points can produce any of the colors in the space, however, a fourth color point adds an additional degree of freedom that allows lumens to be maintained throughout simulations. Each spectral simulation shown in this study are within 1% of lumen and color point targets. Lumen target was chosen arbitrarily at 717 lumens. Color point targets were obtained as central points for each ANSI color bin, obtained from LED datasheets from Lumileds (San Jose, CA, United States) and are as follows:

1,800 K: (*x,y*) = (0.549, 0.408)

2,200 K: (*x,y*) = (0.502, 0.415)

2,700 K: (*x,y*) = (0.458, 0.410)

3,000 K: (*x,y*) = (0.434, 0.403)

3,500 K: (*x,y*) = (0.407, 0.392)

4,000 K: (*x,y*) = (0.380, 0.380)

5,000 K: (*x,y*) = (0.345, 0.355)

6,500 K: (*x,y*) = (0.312, 0.328)

#### Spectral Data

450 nm royal blue LED data was obtained from Lumileds was translated to 410, 420, 430, 440, 460, 470, 480, 490, and 500 nm data for this simulation. Phosphor data was obtained from Merck EMD (Darmstadt, Germany). Phosphors were selected to have longer wavelength while having capability to meet the desired color points within the CIE 1931 color space, which is used to determine white color points. This was done to minimize effect of phosphor on melanopic content. Phosphors have peak wavelengths at 547 nm (YYG-547-210), 585 nm (OGA-585), and 660 nm (YYG-660). Spectra and resulting color points for each component described in this section are plotted for reference in Supplementary Figures 3, 4. Color points are as follows:

410 nm LED: (*x,y*) = (0.1704, 0.0191)

420 nm LED: (*x,y*) = (0.1668, 0.0206)

430 nm LED: (*x,y*) = (0.1627, 0.0236)

440 nm LED: (*x,y*) = (0.1570, 0.0300)

450 nm LED: (*x,y*) = (0.1491, 0.0423)

460 nm LED: (*x,y*) = (0.1370, 0.0666)

470 nm LED: (*x,y*) = (0.1169, 0.1166)

480 nm LED: (*x,y*) = (0.0857, 0.2183)

490 nm LED: (*x,y*) = (0.0509, 0.3943)

500 nm LED: (*x,y*) = (0.0415, 0.6034)

YYG-547-210 Phosphor: (*x,y*) = (0.4512, 0.5326)

OGA-585 Phosphor: (*x,y*) = (0.5581, 0.4410)

YYG-660 Phosphor: (*x,y*) = (0.686, 0.314)

#### Working Color Space Analysis

Combinations of color points for 450 nm LED and three phosphors are bounded in Supplementary Figure 5 to show possible combinations of colors possible with this color mix, creating a “working color space”. ANSI color bins are also plotted for reference. These ANSI bins are the accepted areas by which a manufacturer can specify a white for a given color temperature. This “working color space” exercise is repeated for 410, 490, and 500 nm monochromatic peaks, shown in Supplementary Figures 6–8, respectively. From these charts, we see that not all color temperatures fall within the working spaces. Most notably, 500 nm cannot achieve any of the ANSI color bins from 6,500 to 2,700 K and was removed from analysis.

#### Spectral Optimization for Day and Night

**FIGURE 2:**
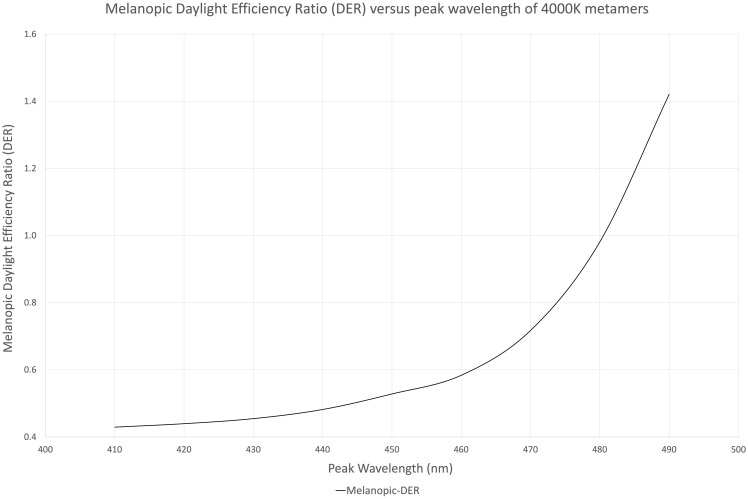
Melanopic daylight efficiency ratio (DER) of the nine 4,000 K spectral metamers from Figure 1 organized by their peak blue emission on the X-axis (410–490 nm).

Figure 1 illustrates nine (9) different metamers of light that produce the same 4,000°K white light with the same lumens. Each metamer has its peak blue wavelength shifted by 10 nm, from 410 to 490 nm. These spectra were evaluated for melanopic DER, shown in Figure 2. We see a DER of a 450 nm peaked 4,000 K light of 0.53, where a 410 nm peaked 4,000 K light has a DER of 0.43. This is a 19% reduction in melanopic DER by moving from a standard 450 nm blue to a 410 nm blue LED. A 490 nm peaked 4,000 K light has a DER of 1.42, a 2.7-fold increase in melanopic DER. Thus spectral optimization can be had for both day and night, however, more advantage might be gained from daytime spectral optimization.

It is important to consider how each of these spectra might render colors. Most of these metamers will not achieve color rendition characteristics needed for commercial viability and thus represent what is possible, but not practical. These metamers simply drive direction for spectrum strategies for day and night. When we control for a minimum color rendering index (CRI) of 80, the melanopic DER of a color-corrected 490 nm peaked 4,000 K spectrum falls to 0.83, compared to a 450 nm peaked spectrum that has a melanopic DER of 0.59. An example of this spectrum is shown in Supplementary Figure 9. This is a much more modest improvement of 41% in melanopic DER over the standard LED. Moreover, a 410 nm peaked spectrum only has a melanopic DER of 0.58, a 2% reduction compared to the standard LED. The trend can be seen in Supplementary Figure 10. Maximum benefit of 410 nm peak LED is gained at 2,700 K, where 410 nm 2,700 K spectrum has a melanopic DER of 0.33 compared to a standard LED 0.41, a 20% reduction. A larger reducation can be had with warmer color temperatures, such as 1,800 K (melanopic DER = 0.18) or 2,200 K (melanopic DER = 0.31), a 56 and 25% reduction, respectively.

On the colder color temperature end, standard 450 nm LED showed a melanopic DER of 0.73 and 0.86 for 5,000 and 6,500 K, respectively, an increase of 23 and 45%, respectively, over 4,000 K standard 450 nm LED. 490 nm peaked LED showed a melanopic DER of 0.93 and 1.12 for 5,000 and 6,500 K, an increase of 12 and 34% over the color-corrected 490 nm peaked 4,000 K LED spectrum. However, color-corrected 490 nm peaked 5,000 K had a 27% increase in melanopic DER over standard 450 nm 5,000 K LED and color-corrected 490 nm peaked 6,500 K had a 30% increase in melanopic DER over standard 450 nm 6,500 K LED.

Thus, spectrum has a larger impact than color temperature when striving for high melanopic DER and color temperature has a larger impact than spectrum when striving for low melanopic DER. Of course, both color temperature and spectrum should be employed whenever possible.

### Other Biological Models

Other biological models have been proposed. The Circadian Stimulus (CS) Model has a peak sensitivity at 460 nm rather than 490 nm and includes interactions from rods and cones into the ipRGC response for color temperatures greater than 3,500 K that results in a subtraction in the green wavelengths. For color temperatures of 3,500 K and warmer, CS uses melanopsin as its sensitivity function (Figueiro et al., 2008). Circadian Potency Spectral Sensitivity (CPSS) has been proposed, suggesting a peak sensitivity at 477 nm with a narrower sensitivity band than melanopsin (Moore-Ede et al., 2020).

Each of the nine (9) 4,000 K metamers from Figure 1 were applied to CS for color temperatures greater than 3,500 K (CS Cool) and CPSS (Supplementary Figure 11) and evaluated for relative biological potency relative to itself (Supplementary Figure 12) at 410 nm peaked 4,000 K. CS has the least amount of biological potency with the 420 and 430 nm spectra, but still we see a fivefold increase with the non-color-corrected 490 nm peaked LED spectrum compared to the 410 nm LED spectrum. This is a surprising result for a model with a peak sensitivity at 460 nm. The reason is longer wavelength blues stimulate the *Y* tristimulus color matching function. This has to be counter-balanced by less green phosphor, which is primarily in the subtraction portion of the CS sensitivity curve.

The CPSS also has a minimum potency with the 420 nm LED spectrum, but still sees a 4.2-fold increase in biological potency from a non-color-corrected 490 nm peaked LED spectrum compared to a non-color-corrected 410 nm LED spectrum. Again this is a surprising effect from a curve with spectral sensitivity peak at 477 nm. This is due to the shape of the 490 nm LED, which has tremendous overlap of the 480 nm LED and 470 nm LED spectra.

In other words, the 490 nm peak is so large that it overshadows shorter wavelength LEDs. This large peak is required because the tristimulus color matching functions are much less sensitive at 490 nm compared to shorter wavelengths. In other words, the biological potential of a 490 nm peaked spectrum found in this analysis may not be due to the peak sensitivity of melanopsin at 490 nm, but rather due to the insensitivity of the tristimulus functions at longer wavelengths, allowing for more total radiant energy for the same visual lumens and perceived color.

## Other Key Variables

While spectrum and color temperature of the light sources are of primary importance, there are secondary considerations that pertain to the biological relevance of the delivery of light. How the light exits the luminaire and how light bounces off the walls can contribute to biological relevance.

### Geometry of the Built Environment on Vertical Illuminance

Commercial buildings often have a grid ceiling, often referred to as a drop ceiling, that include an array of lighting and heating, ventilation, and air conditioning (HVAC) systems integrated inside. This approach in architecture is the most cost-effective for wiring lighting and routing air ducts. This approach points lights downward toward desks and floors to optimize luminance and contrast of objects to be seen. This provides efficiency for visual applications, but lacks direct illumination into the occupants eyes, making this approach inefficient for providing the vertical illumination necessary for circadian daytime exposure.

Current lighting standards and recommendations for lighting are solely placed on the visual criteria as it relates to completing tasks that occur on a horizontal plane (i.e., on a desk). For example, an office may require 300 lumens per square meter (lux) to fall on the desk but make no criteria for what falls on the eyes of the occupants. Key factors related to how much vertical light you can achieve from these common luminaires are room geometry and wall reflectance. The ratio of wall area to floor area is referred to as the room cavity ratio (RCR), as this quantifies the opportunity for light to bounce off a vertical surface and redirect its weight from the horizontal plane to the vertical plane.

$$\text{RCR}=2.5\times \frac{\text{Total}\text{wall}\mathrm{area}\mathrm{above}\mathrm{work}\mathrm{plane}}{\text{Floor}\mathrm{area}}$$

Larger RCR values correspond to more wall area per floor area. For example, a private office may have an RCR of 5, while an open plan office has a RCR closer to 1.

Simulation of various RCRs at 50% wall reflectivity was achieved using AGi32 lighting design software from Lighting Analysts (Littleton, CO, United States) with typical lighting fixtures. Space types for the simulations included the following:

Open Office (46′ × 86′ × 9′) with recessed light fixtures, RCR = 0.8

Open Office (46′ × 86′ × 9′) with suspended light fixtures 18″ below the ceiling, RCR = 1.1

Classroom (20′ × 30′ × 9′) using recessed fixtures, RCR = 2.1

Classroom (20′ × 30′ × 9′) with suspended light fixtures 18″ below the ceiling, RCR = 2.7

Break Room (12′ × 20′ × 9′) with recessed light fixtures, RCR = 3.3

Break Room (12′ × 20′ × 9′) with suspended light fixtures 18″ below the ceiling, RCR = 4.3

Office/Conf. Room (12′ × 12′ × 9′) with recessed light fixtures, RCR = 4.2

Office/Conf. Room (12′ × 12′ × 9′) with suspended light fixtures 18″ below the ceiling, RCR = 5.4

Each space type outlined above also used the following luminaire types/manufacturers:

Recessed Downlights / Ledra Alphabet NU3RD (Tustin, CA, United States)

Recessed Wall Washer / Ledra Alphabet NU3RW (Tustin, CA, United States)

Recessed 2 × 2 Troffer / Pinnacle Lucen (Denver, CO, United States)

Recessed 2 × 4 Troffer / Pinnacle Lucen (Denver, CO, United States)

Direct Linear Pendant / Pinnacle Edge3 (Denver, CO, United States)

Indirect Linear Pendant / Axis Beam4 (Lasalle, QC, Canada)

Direct / Indirect Linear Pendant / Axis Surround Lite (Lasalle, QC, Canada)

Direct / Indirect Circular Area Pendant / Prudential P4000 (Los Angeles, CA, United States)

An example of these AGi32 calculations is shown in Supplementary Figures 13–15. Supplementary Figure 16 illustrates the benefit in vertical to horizontal ratio using different fixture types versus RCR. In summary, an open plan office will provide 50 vertical lux at the occupant’s eyes for every 100 lux on the desktop. Whereas a private office will provide 80 vertical lux at the occupant’s eyes for every 100 lux on the desk surface, a 60% boost in vertical illumination.

These calculations were repeated for 70% wall reflectivity and 90% wall reflectivity. A boost in vertical to horizontal ratio was not observed due to wall reflectivity, however, a boost in total illuminance was observed. Supplementary Figures 17, 18 illustrate these vertical illumination benefits of higher wall reflectivity as a function of RCR. These Figures illustrate that vertical illumination benefits were minor for small RCRs, but much stronger for larger RCRs. In other words, increasing wall reflectivity in a large open plan office will yield a meager benefit, but increasing wall reflectivity in a private office will have a significant benefit to vertical illumination.

### Luminaire Light Distribution

Light distribution is another characteristic that plays a critical role in achieving vertical illuminance. Pendants are a type of luminaire that are mounted to the ceiling and suspended in the space. Some pendants are fully luminous, such that it distributes light in all directions. We evaluated the Purelight Round Luminous pendant from Selux (Highland, NY, United States) that focus the majority of their energy downward toward the task plane. Our AGi32 analysis showed that these luminous surfaced pendants provided 22% more vertical illumination on average for the same amount of light on the task plane.

Different lighting strategies can also be applied at night. Typical LED “Edison-type” light bulbs do not have the same isotropic distribution as a standard incandescent light source. These light bulbs direct the majority of the light in the direction opposite of screw-in base. When applied into a standard table lamp with the screw base downward, light is directed onto the 80% reflective ceiling. Mirror light bulbs are fairly common and designed to reduce direct glare and can be used in place of these standard Edison LED bulbs to redirect the light from the 80% reflective ceiling toward a much lower reflectance floor. A study was conducted in a 14′ × 10′ × 8′ (L × W × H) room, where a single table lamp was illuminated in a corner of the room using both a regular Edison-type light bulb and a mirror type light bulb. Vertical illumination was measured facing the lamp from 2′ to 10′ away using both a standard LED lamp and a mirror lamp of the same lumen output. Measurements are provided in supplemental Supplementary Figure 19 which shows that this technique yielded a 35% reduction in vertical illumination, while still maintaining light availability.

### Controls

Controls play an integral role in biologically relevant lighting. Controls should be configured to create brighter days and darker nights on a consistent and predictable cadence. This can be set to be in phase with the solar cycle or can be set to a specific time, such as 6 a.m. to 8 p.m. The later maybe preferred at higher latitudes. At a bare minimum, this control system would comprise of automatic dimmers that increase and decrease intensity according to time. However, a biologically relevant lighting system should consider intensity, spectrum, and distribution. Intensity and spectrum should be tied together to maximize day versus night delineation. Blending between a brighter intensity of blue enriched spectrum with peak emission at 490 nm of the highest accepted color temperature and dimmer intensity of blue depleted spectrum with peak emission between 410 and 450 nm of lowest acceptable color temperature. Further day/night delineation value can be obtained by including spatial distribution. Nighttime scenes should include no indirect light (lights pointed at the ceiling) and should have direct light (lights pointed at the task) with blue depleted spectrum of minimal intensity to achieve necessary tasks. Daytime scenes should include direct and indirect light, both with blue enriched spectrum with peak emission at 490 nm of highest accepted color temperature and of highest accepted intensity both in terms of comfort and energy constraints.

## Personal Circadian Lighting Devices

One final strategy to help provide biologically relevant lighting within a space is the use of personalized devices that can supply supplemental vertical lighting. These personalized devices could be located relatively close to the occupant and primarily provide vertical illumination, for example something akin to a light box or a table lamp. These types of devices could conceivably add 200 melanopic EDI or more at the eye of the occupant and could be tailored to the needs of an individual. Ideally, this type of intervention would be automated to dynamically transition between day and night illumination throughout the day.

## Daylighting

The data presented here provides quantified strategies for implementing biologically brighter days and darker nights into the built environment. While not quantified in this analysis, daylight exposure is best for providing circadian benefits (Knoop et al., 2020), but it should be noted that vertical light exposure from windows drops off dramatically with distance from said window. Skylights and daylight harvesting strategies are encouraged to bring that daylight deeper into the space to maximize its benefits.

## Compounding the Strategies for Brighter Days and Darker Nights

Standalone strategies for brighter days and darker nights are outlined in this article and while some individual interventions may not seem like they apply a space or application, the combination of these strategies compound their benefits to create a biologically relevant lighting environment.

For example, a person spends their day in the office under standard 3,500 K LED lighting with 300 lux at the task plane and 150 lux at the eye. A standard 3,500 K LED spectrum has a melanopic DER of about 0.51, thus the melanopic EDI at the eye is 76.5. The same person spends their evening in a home with luminaires populated with 2,700 K LED light bulbs. These luminaires provide approximately 50 lux at the eye. 2,700 K LED spectrum has a melanopic DER of about 0.4, thus a nighttime melanopic EDI of 20. This is a typical example of how light is biologically too dim for daytime use (76.5 melanopic EDI) and too bright for evening (20 melanopic EDI), with a day-to-night ratio of 3.8:1. Its not certain if more benefit would be gained from brighter days or darker nights, it is good practice to improve both and increase that day-to-night ratio.

At the office, the simple incorporation of a spectrally optimized daytime light source with a slightly cooler color temperature of 4,000°K will lead to a 58% boost in daytime biological potency. This would increase 76.5 melanopic EDI from the example to 121 melanopic EDI. While at home, incorporating spatially optimized light bulbs with a slightly warmer color temperature of 2,200°K lighting provides a 60% reduction in nighttime biological potency. This would decrease 20 melanopic EDI from the example to eight melanopic EDI. Combining these two strategies would increase the day-to-night ratio in the example from 3.8:1 to 15.1:1. This is a 392% increase in the delineation of daytime versus nighttime.

The proposed goal is to combine as many of these individual strategies and put them into practice (when possible) while maintaining excellent light quality, appropriate design aesthetic, and achieving user/occupant compliance. These strategies are itemized here:

**Table:** 

Daytime strategy	Standalone benefit
(1) Color-corrected spectrally optimized daytime spectrum	∼ (+41%)
(2) Colder color temperatures	∼ (+10%) per 500 K
(3) Spatially optimized luminaire	∼ (+22%)
(4) Private office	∼ (+60%)
(5) Private office with highly reflective walls	∼ (+150%)
(6) Personal circadian luminaire	+200 melanopic EDI
**Nighttime strategy**	**Standalone benefit**
(1) Warmer color temperatures	∼(−15%) per 500 K
(2) Spatially optimized luminaire	∼(−35%)
(3) Spectrally optimization	Up to −20%

## Data Availability Statement

The raw data supporting the conclusions of this article will be made available by the authors, without undue reservation.

## Author Contributions

All spectral analysis, industry perspective, data collection, and analysis were done by RS. Lighting design simulation was done by EV, an employee of BIOS lighting.

## Conflict of Interest

RS is a founder of BIOS Lighting, a for-profit lighting manufacturer. EV is currently employed by the company BIOS Lighting, a for-profit company.

## Supplementary Material

Supplementary Figure 1 Tristimulus sensitivity according to CIE 1931 color matching functions and melanopsin sensitivity according to CIE S 026.

Supplementary Figure 2 Standard off-the-shelf 6,500 K LED with peak blue emission at 450 nm and broad phosphor excitation emission from 480 to 780 nm compared to tristimulus sensitivity according to CIE 1931 and melanopsin sensitivity according to CIE S 026.

Supplementary Figure 3 Spectral Power Distribution (SPD) for each component used in simulation. **(A)** SPD of 410 nm LED; **(B)** SPD of 420 nm LED; **(C)** SPD of 430 nm LED; **(D)** SPD of 440 nm LED; **(E)** SPD of 450 nm LED; **(F)** SPD of 460 nm LED; **(G)** SPD of 470 nm LED; **(H)** SPD of 480 nm LED; **(I)** SPD of 490 nm LED; **(J)** SPD of 500 nm LED; **(K)** SPD of 547 nm peaked phosphor YYG-547-210; **(L)** SPD of 585 nm peaked phosphor OGA-585; **(M)** SPD of 660 nm peaked phosphor YYG-660.

Supplementary Figure 4 Color plots of each component used in simulation. Color points are labeled A through M, in reference to spectral power distributions of said color plot from Supplementary Figure 3.

Supplementary Figure 5 Working space of 450 nm LED (E), 547 nm peaked phosphor YYG-547-210 (K), 585 nm peaked phosphor OGA-585 (L), and 660 nm peaked phosphor YYG-660 (M). Any color points within the triangle plotted here can be created. ANSI color temperature bins for 6,500, 5,000, 4,000, 3,500, 3,000, and 2,700 K white light are plotted for reference.

Supplementary Figure 6 Working space of 410 nm LED (A), 547 nm peaked phosphor YYG-547-210 (K), 585 nm peaked phosphor OGA-585 (L), and 660 nm peaked phosphor YYG-660 (M). Any color points within the triangle plotted here can be created. ANSI color temperature bins for 6,500, 5,000, 4,000, 3,500, 3,000, and 2,700 K white light are plotted for reference.

Supplementary Figure 7 Working space of 490 nm LED (I), 547 nm peaked phosphor YYG-547-210 (K), 585 nm peaked phosphor OGA-585 (L), and 660 nm peaked phosphor YYG-660 (M). Any color points within the triangle plotted here can be created. ANSI color temperature bins for 6,500, 5,000, 4,000, 3,500, 3,000, and 2,700 K white light are plotted for reference.

Supplementary Figure 8 Working space of 500 nm LED (J), 547 nm peaked phosphor YYG-547-210 (K), 585 nm peaked phosphor OGA-585 (L), and 660 nm peaked phosphor YYG-660 (M). Any color points within the triangle plotted here can be created. ANSI color temperature bins for 6,500, 5,000, 4,000, 3,500, 3,000, and 2,700 K white light are plotted for reference.

Supplementary Figure 9 Blended spectrum that achieves a minimum of 80 CRI and maximizes energy efficiency and melanopic daylight efficiency ratio (DER).

Supplementary Figure 10 Melanopic DER versus color temperature of color-corrected optimized daytime spectrum (dashed line), color-corrected optimized nighttime spectrum (dash-dot line), and standard LED (solid line).

Supplementary Figure 11 Nine spectral metamers of light, each with different blue light peaks. 410 nm peaked metamer (purple), 420 nm peaked metamer (green), 430 nm peaked metamer (yellow), 440 nm peaked metamer (red), 450 nm peaked metamer (royal blue), 460 nm peaked metamer (orange), 470 nm peaked metamer (black), 480 nm peaked metamer (dark gray), and 490 nm peaked metamer (sky blue), from Figure 1, are overlayed on top of melanopsin sensitivity function (gray–solid) according to CIE S 026, CS cool sensitivity function (gray–small dash) and CPSS (gray–dash dot). Each metamer produces visually the same 4,000 K color of light at the same lumens according to the tristimulus functions.

Supplementary Figure 12 Relative weights of m-DER, CS, and CPSS of the nine spectral metamers from figure S10 (Y-axis) versus peak blue wavelength of metamer (X-axis). Weights are relative to themselves at the 410 nm peaked metamer.

Supplementary Figure 13 Elevation view of AGi32 lighting simulation software and lighting calculation points of an open office layout. Calculation points are horizontal (with lines pointed up from points) and vertical (with lines pointed left from points). Blue lines reference building structure. Black lines reference furniture.

Supplementary Figure 14 Plan view of AGi32 lighting simulation software and vertical lighting calculation points of an open office layout. Calculation points are vertical with lines pointed in direction of view. Blue lines reference building structure. Black lines reference furniture. Red lines reference light fixtures.

Supplementary Figure 15 Rendering of isometric view using AGi32 lighting simulation software.

Supplementary Figure 16 Vertical to Horizontal light ratio versus room cavity ratio with 90% reflective painted walls for of downlight luminaires (gray–solid), direct pendent luminaires (black–solid), wall washer luminaires (gray–dashed), indirect/direct pendent luminaires (black–dashed), indirect only pendent luminaires (gray–dash dot), and recessed general illumination luminaires (black–dash dot).

Supplementary Figure 17 Boost in vertical melanopic EDI versus RCR of spaces with 90% wall reflectivity compared to spaces with 50% wall reflectivity of downlight luminaires (gray–solid), direct pendent luminaires (black–solid), wall washer luminaires (gray–dashed), indirect/direct pendent luminaires (black–dashed), indirect only pendent luminaires (gray–dash dot), and recessed general illumination luminaires (black–dash dot).

Supplementary Figure 18 Boost in vertical melanopic EDI versus RCR of spaces with 70% wall reflectivity compared to spaces with 50% wall reflectivity of downlight luminaires (gray–solid), direct pendent luminaires (black–solid), wall washer luminaires (gray–dashed), indirect/direct pendent luminaires (black–dashed), indirect only pendent luminaires (gray–dash dot), and recessed general illumination luminaires (black–dash dot).

Supplementary Figure 19 Measurements of vertical illumination (lux) at 40″ height for a space with a single table lamp containing a standard LED A19 light bulb “upward lamp” (black–solid) or a A19 light bulb with a mirror coating “downward lamp” (black–dashed) versus distance away from lamp. Measurements taken facing the lamp.
