# Supplementary figures and images for: Biologically Relevant Lighting: An Industry Perspective
Source: Front Neurosci. 2021 Jun 7;15:637221. doi: 10.3389/fnins.2021.637221 (PMC8215265; doi:10.3389/fnins.2021.637221)

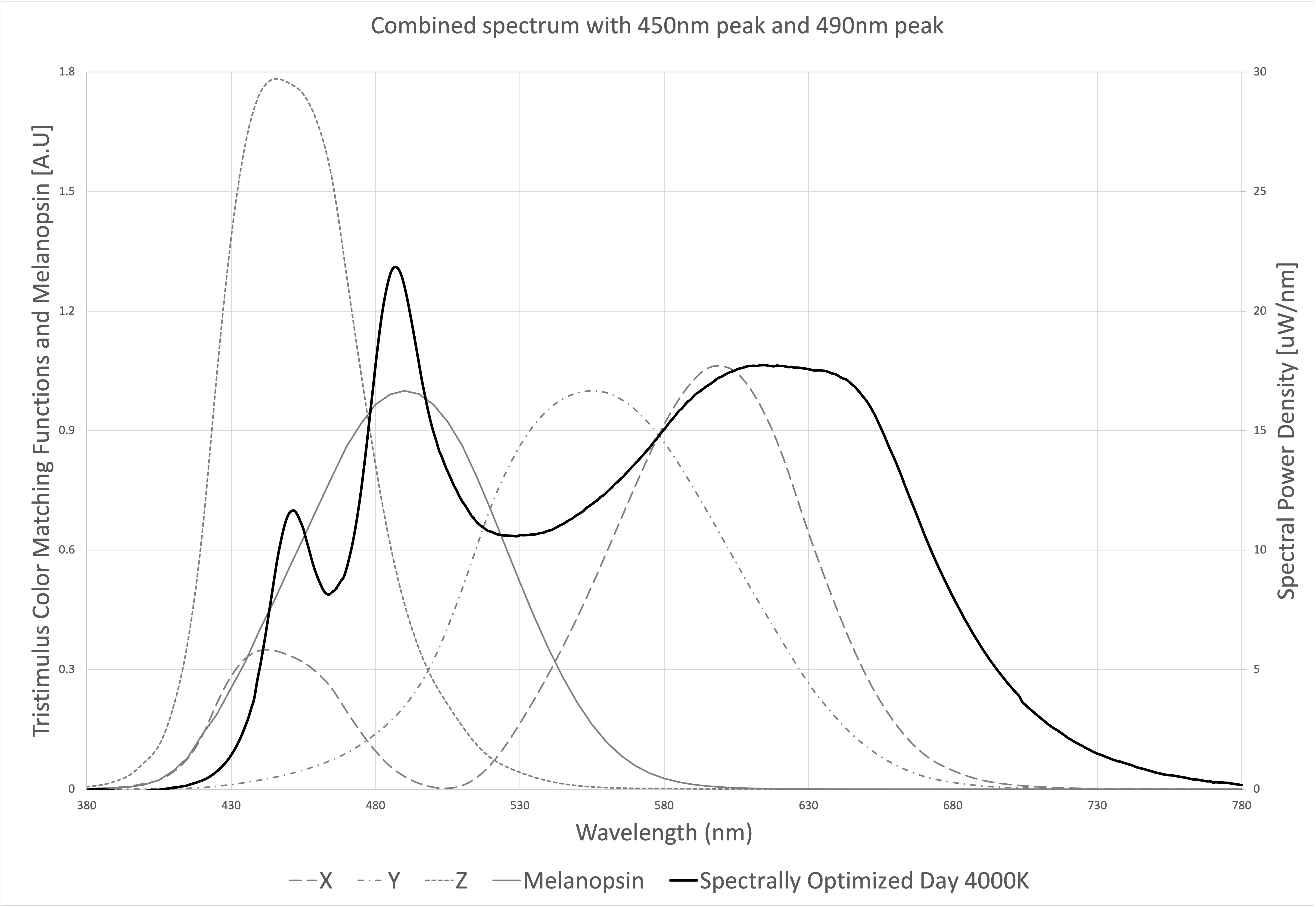

Supplement: Supplementary Figure 1 — Tristimulus sensitivity according to CIE 1931 color matching functions and melanopsin sensitivity according to CIE S 026. [file Data_Sheet_1.ZIP › Figures/Figure S9.jpg]

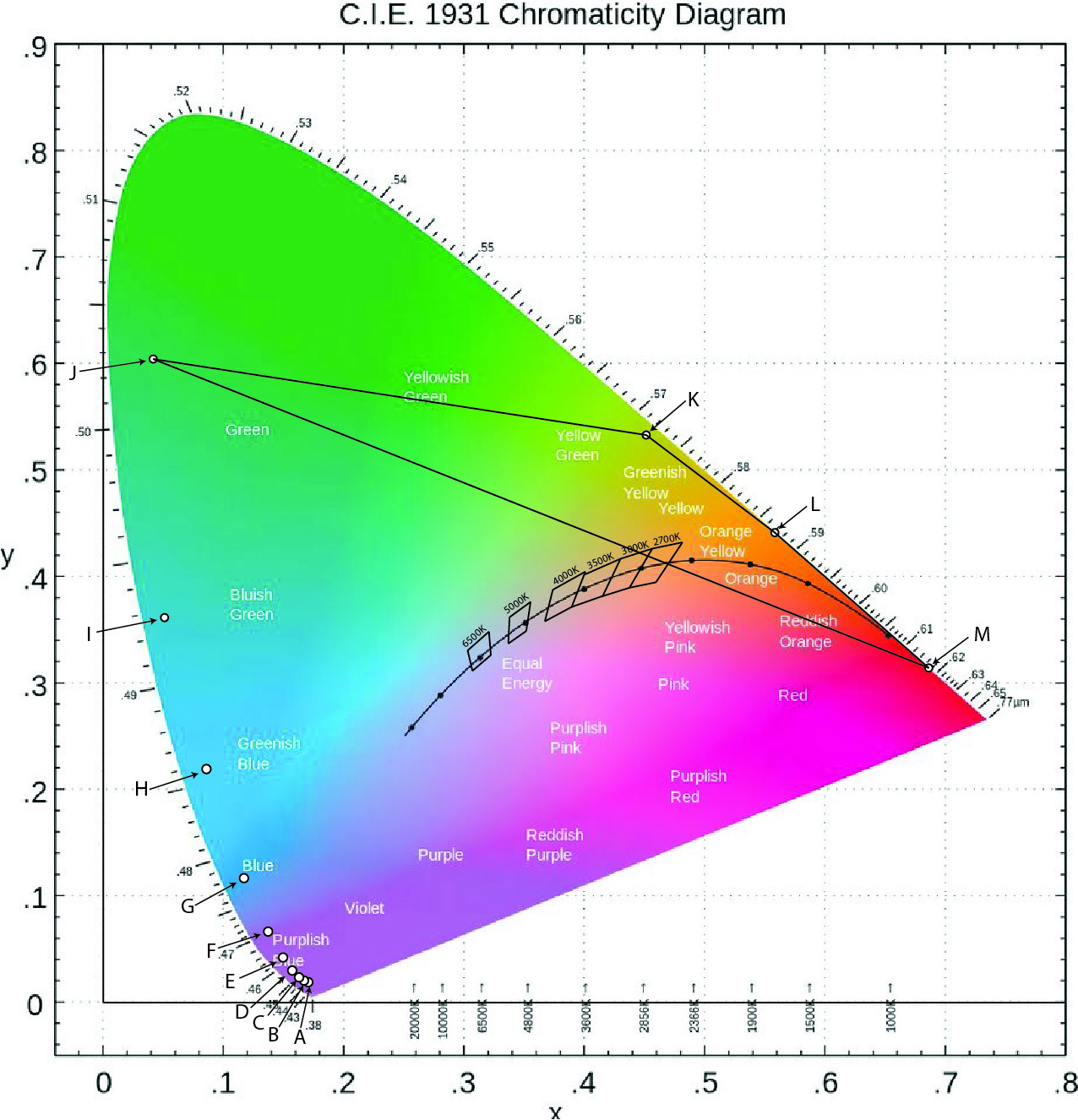

Supplement: Supplementary Figure 1 — Tristimulus sensitivity according to CIE 1931 color matching functions and melanopsin sensitivity according to CIE S 026. [file Data_Sheet_1.ZIP › Figures/Figure S8.jpg]

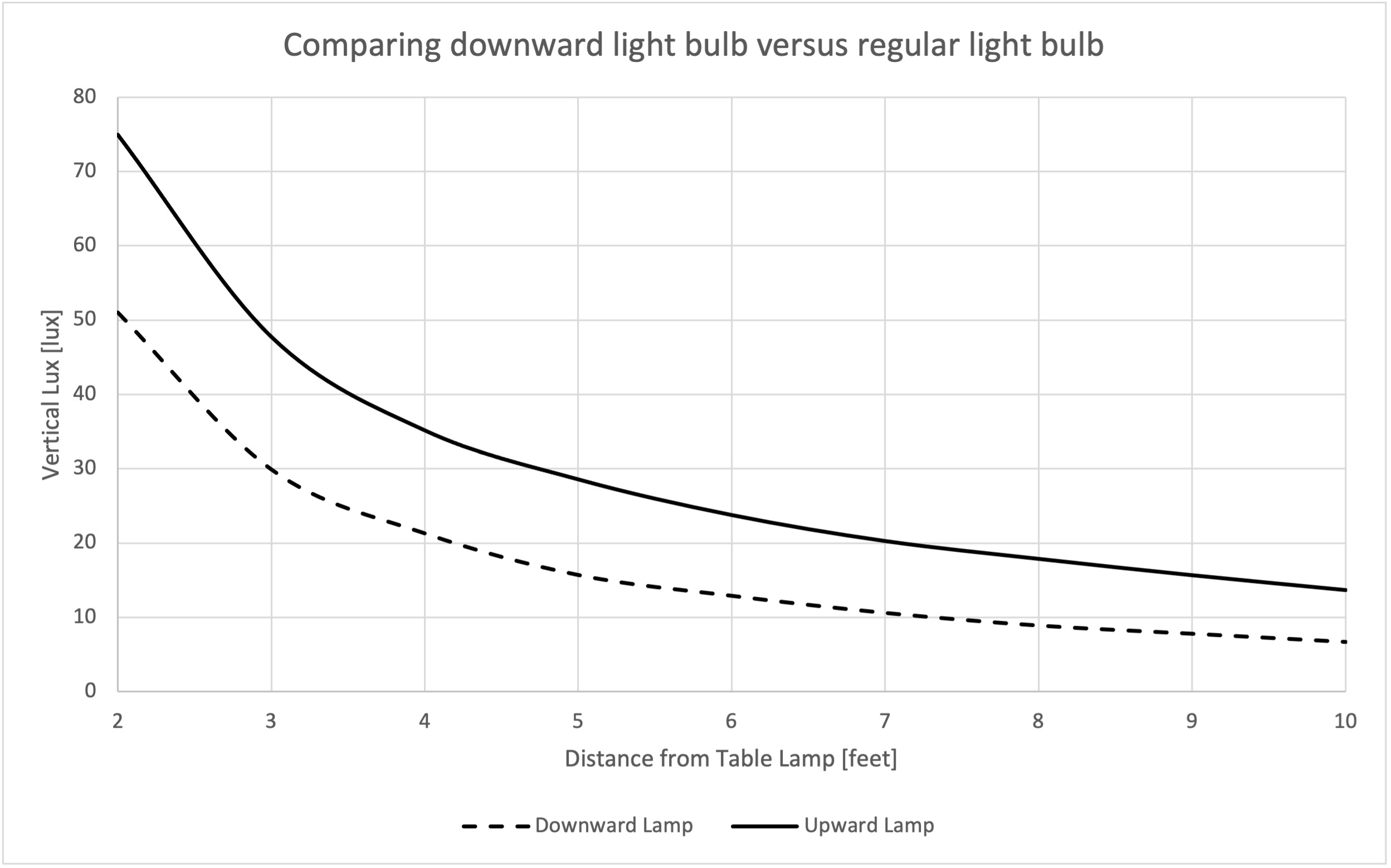

Supplement: Supplementary Figure 1 — Tristimulus sensitivity according to CIE 1931 color matching functions and melanopsin sensitivity according to CIE S 026. [file Data_Sheet_1.ZIP › Figures/Figure S19.jpg]

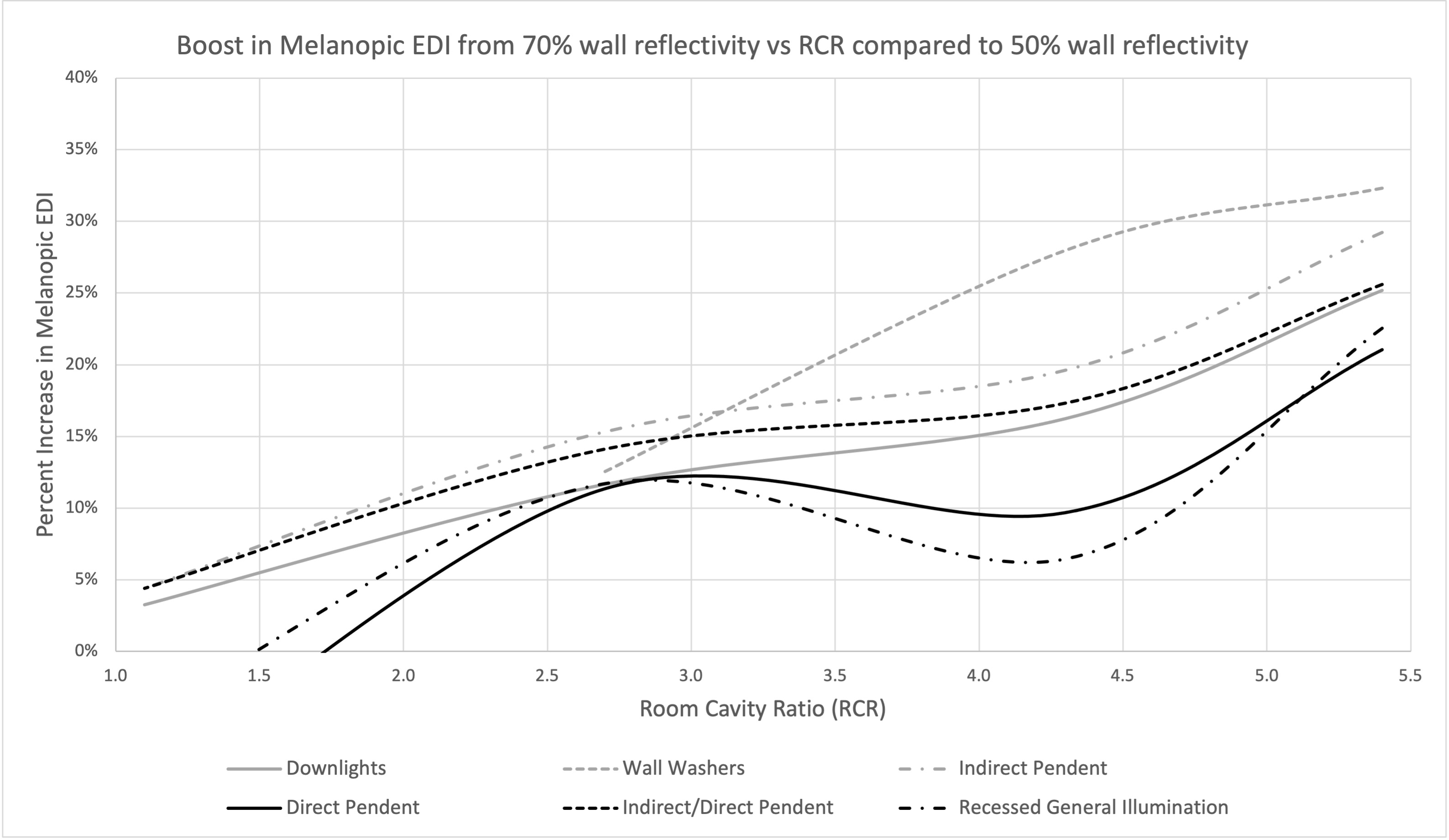

Supplement: Supplementary Figure 1 — Tristimulus sensitivity according to CIE 1931 color matching functions and melanopsin sensitivity according to CIE S 026. [file Data_Sheet_1.ZIP › Figures/Figure S18.jpg]

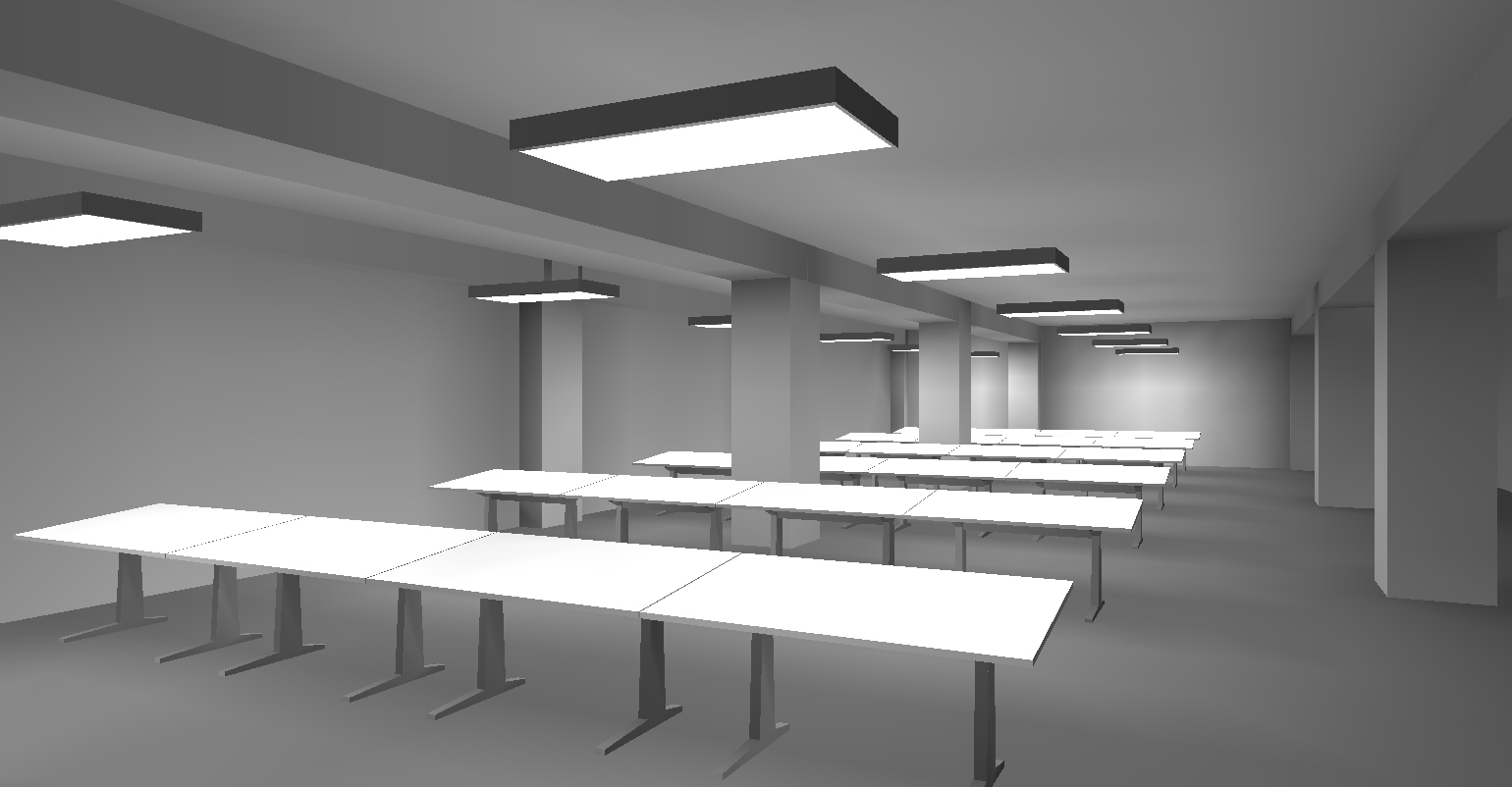

Supplement: Supplementary Figure 1 — Tristimulus sensitivity according to CIE 1931 color matching functions and melanopsin sensitivity according to CIE S 026. [file Data_Sheet_1.ZIP › Figures/Figure S15.jpg]

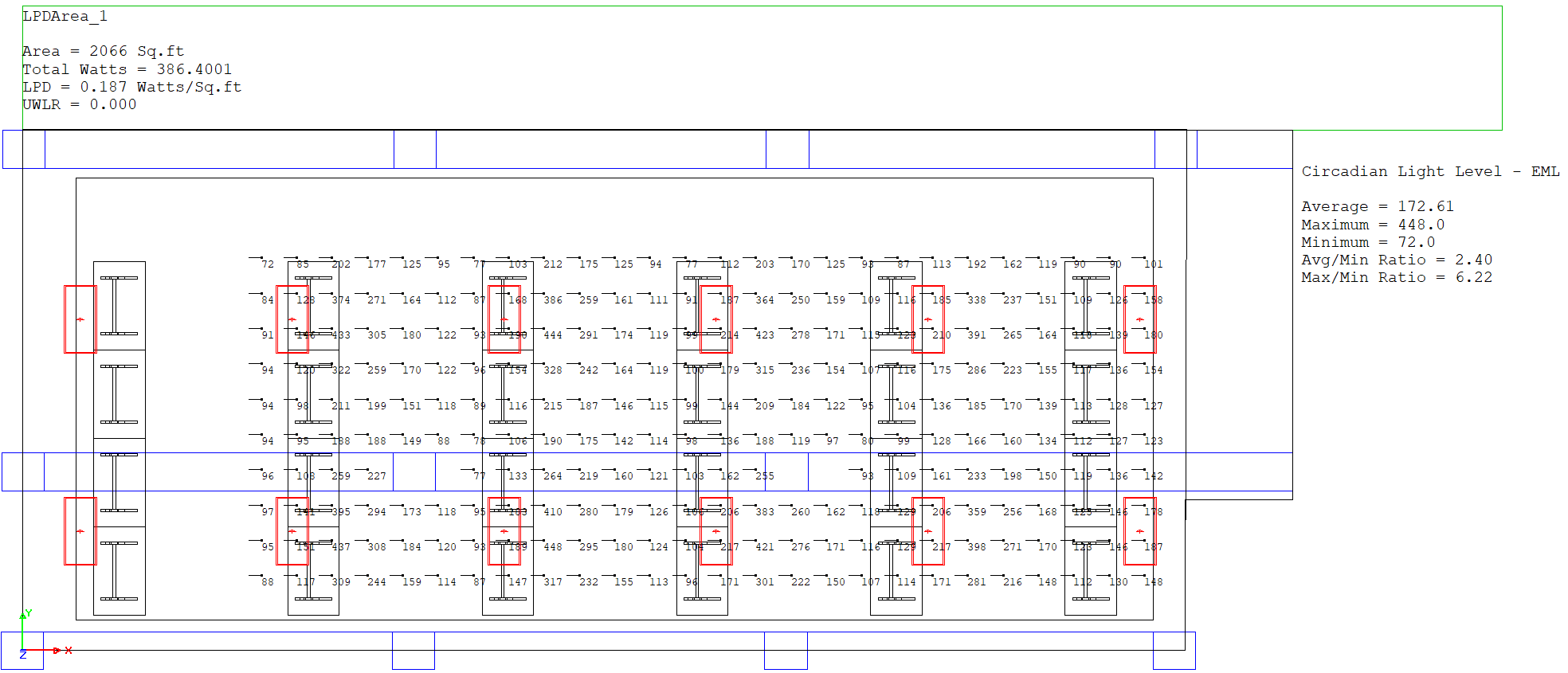

Supplement: Supplementary Figure 1 — Tristimulus sensitivity according to CIE 1931 color matching functions and melanopsin sensitivity according to CIE S 026. [file Data_Sheet_1.ZIP › Figures/Figure S14.PNG]

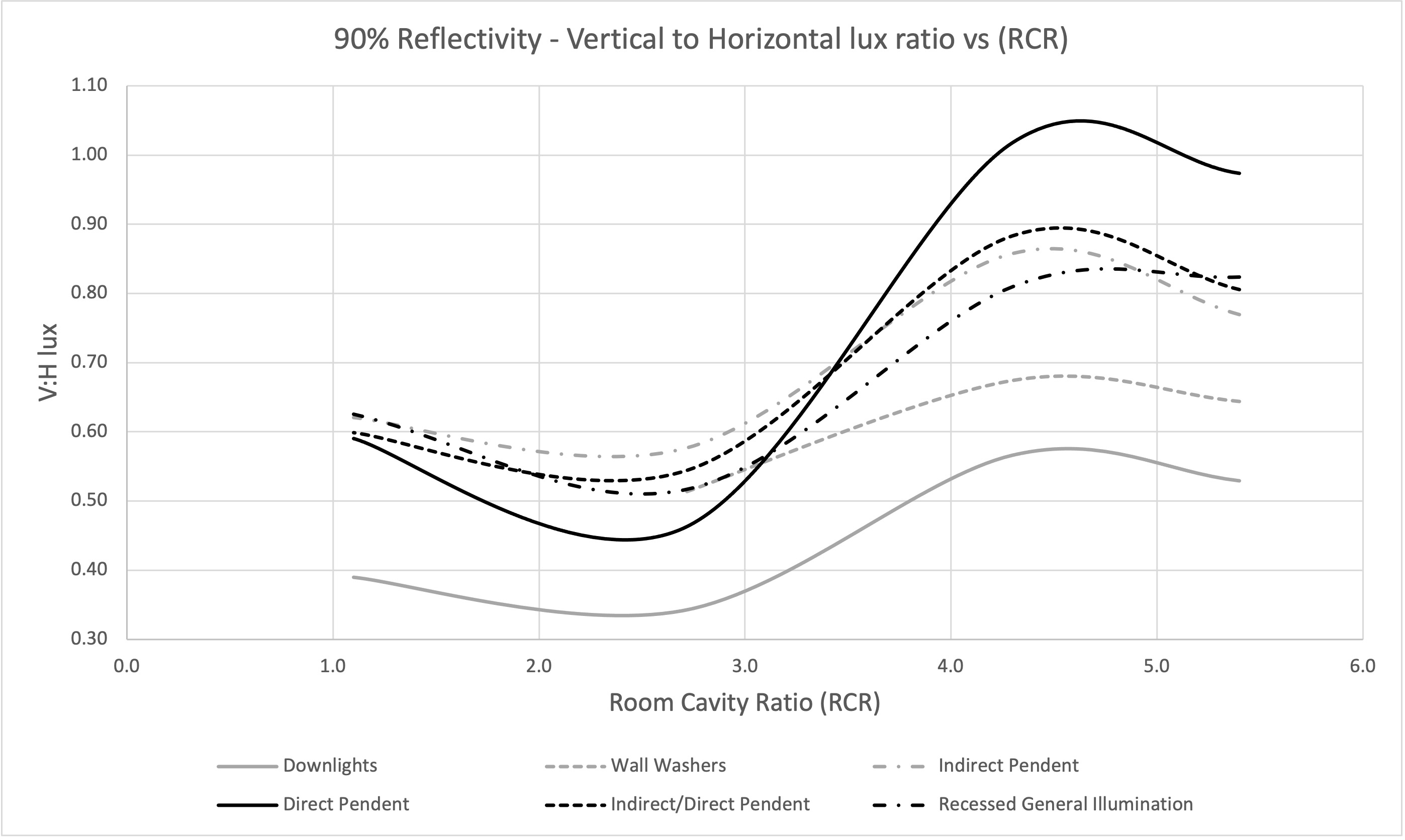

Supplement: Supplementary Figure 1 — Tristimulus sensitivity according to CIE 1931 color matching functions and melanopsin sensitivity according to CIE S 026. [file Data_Sheet_1.ZIP › Figures/Figure S16.jpg]

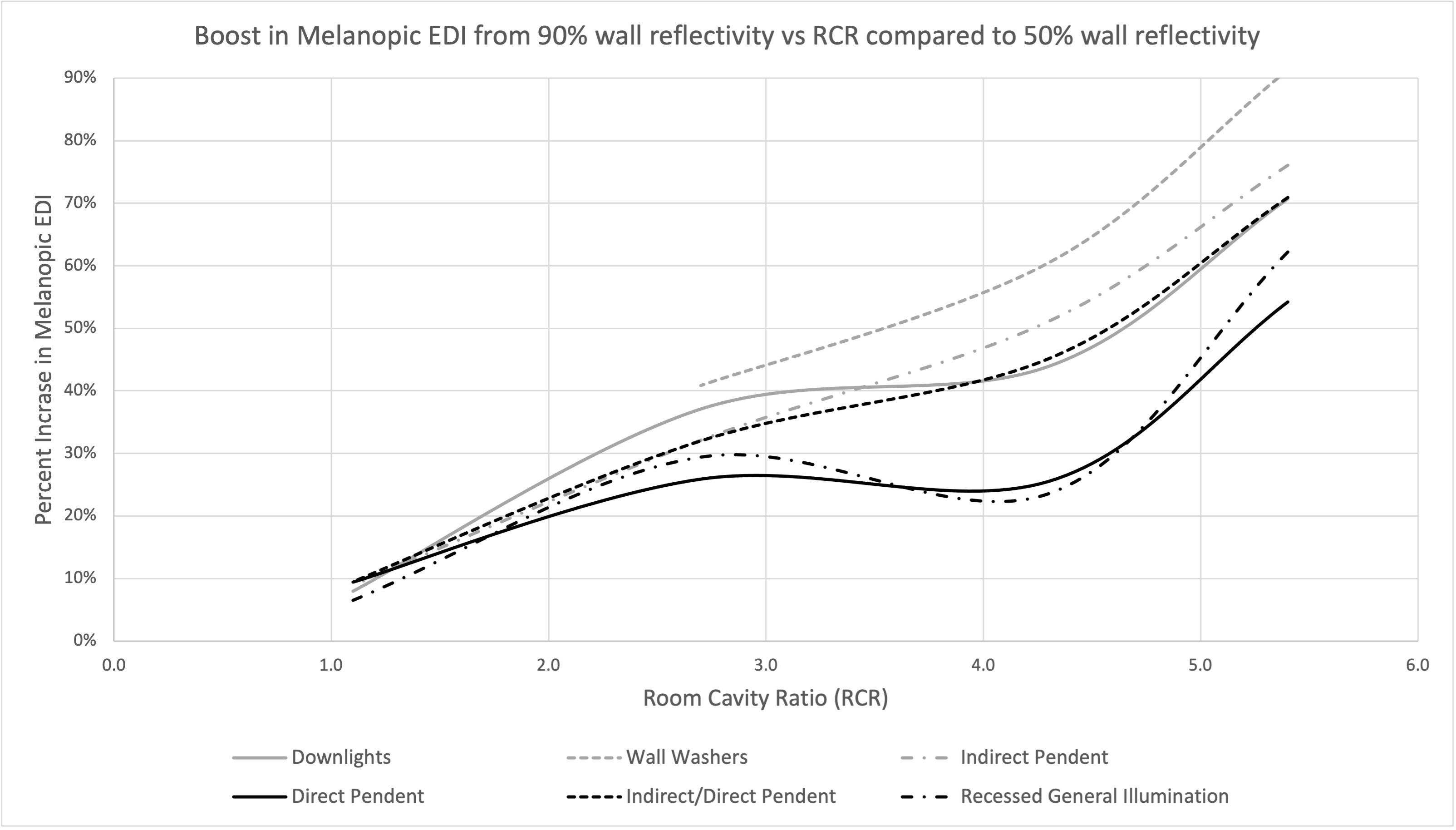

Supplement: Supplementary Figure 1 — Tristimulus sensitivity according to CIE 1931 color matching functions and melanopsin sensitivity according to CIE S 026. [file Data_Sheet_1.ZIP › Figures/Figure S17.jpg]

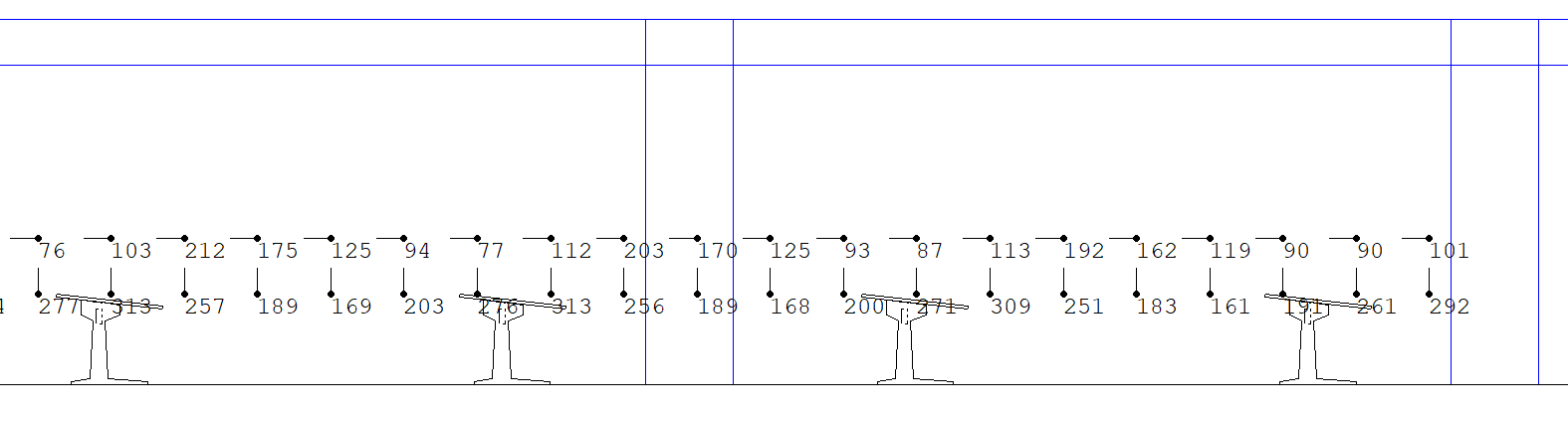

Supplement: Supplementary Figure 1 — Tristimulus sensitivity according to CIE 1931 color matching functions and melanopsin sensitivity according to CIE S 026. [file Data_Sheet_1.ZIP › Figures/Figure S13.PNG]

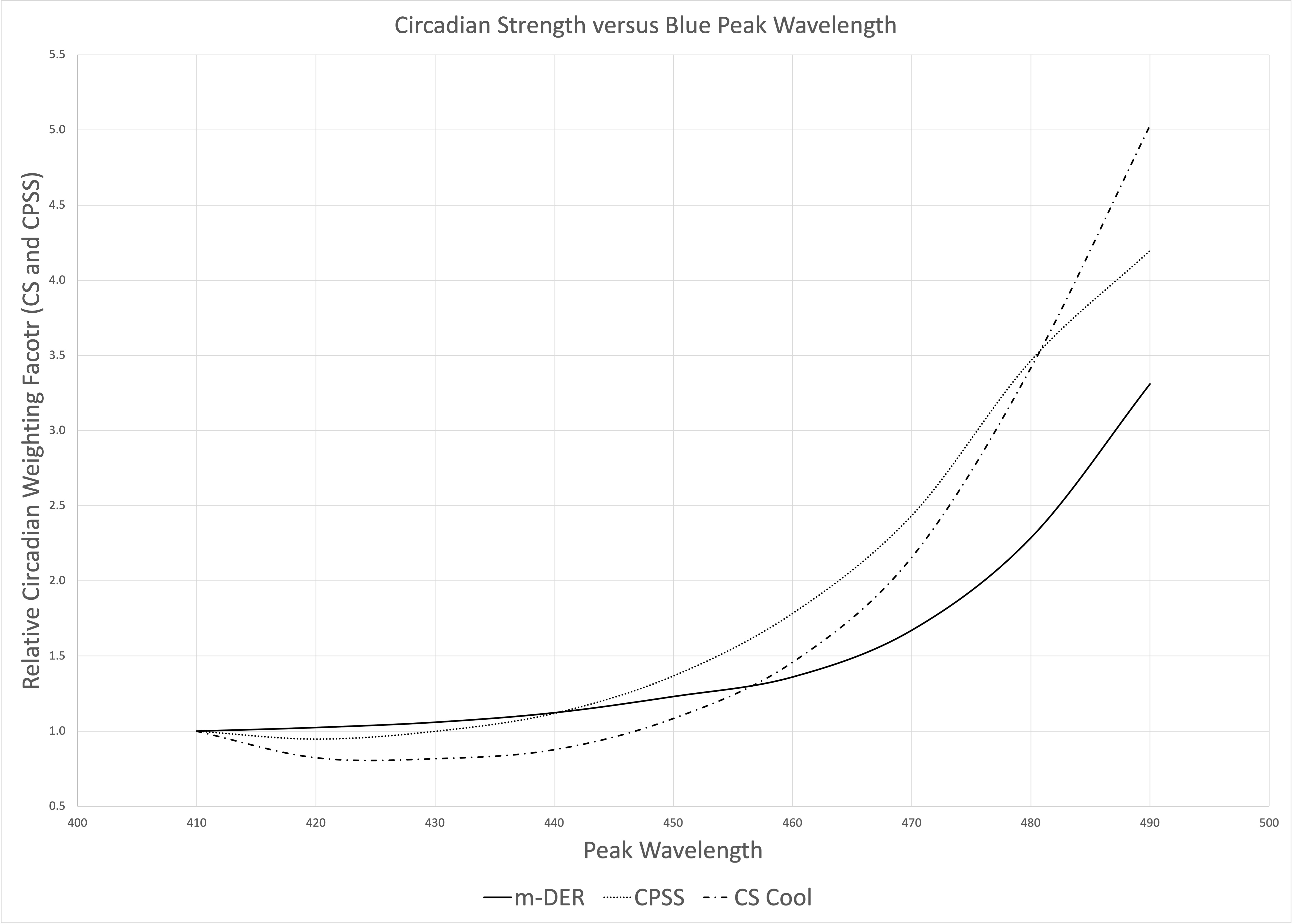

Supplement: Supplementary Figure 1 — Tristimulus sensitivity according to CIE 1931 color matching functions and melanopsin sensitivity according to CIE S 026. [file Data_Sheet_1.ZIP › Figures/Figure S12.jpg]

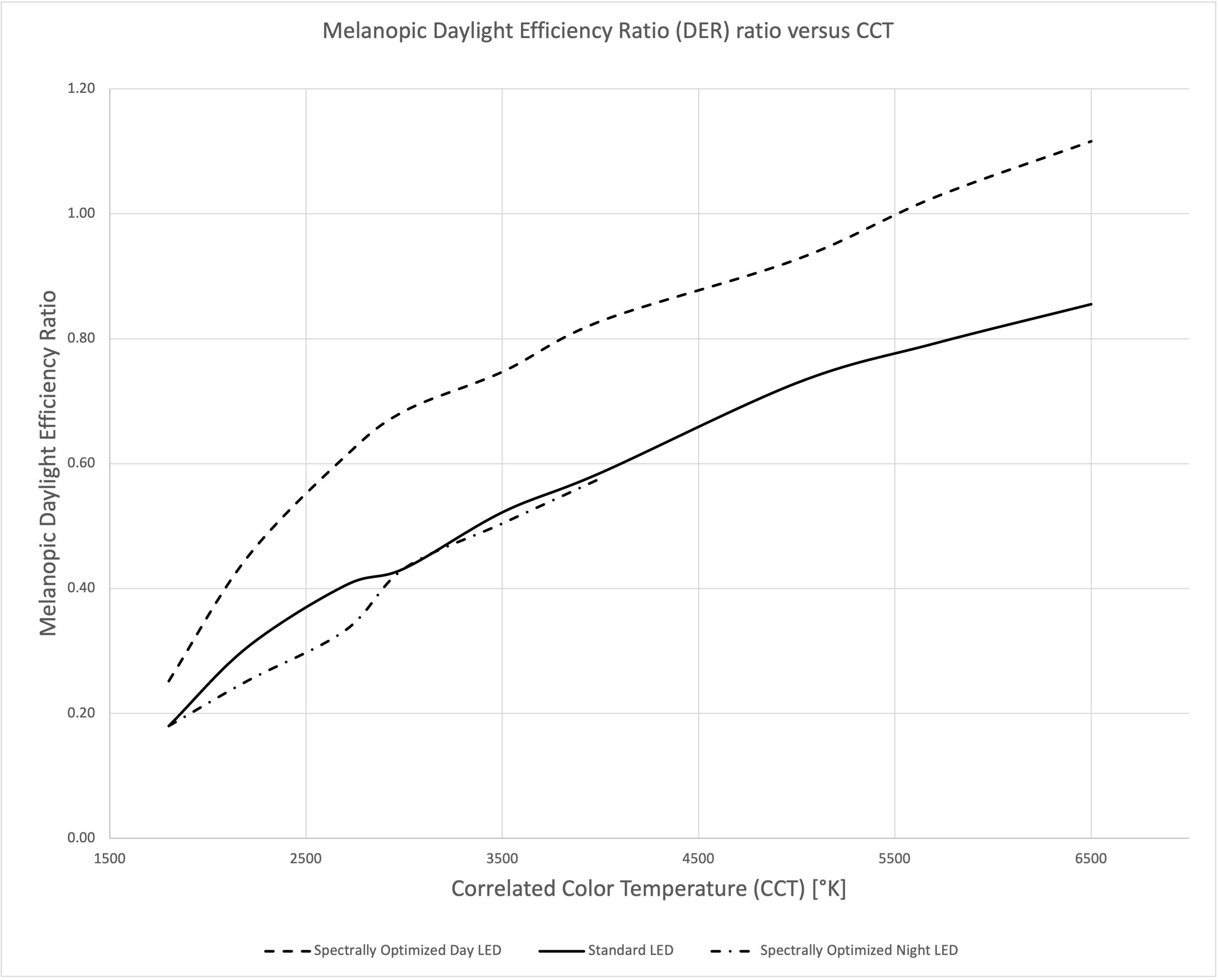

Supplement: Supplementary Figure 1 — Tristimulus sensitivity according to CIE 1931 color matching functions and melanopsin sensitivity according to CIE S 026. [file Data_Sheet_1.ZIP › Figures/Figure S10.jpg]

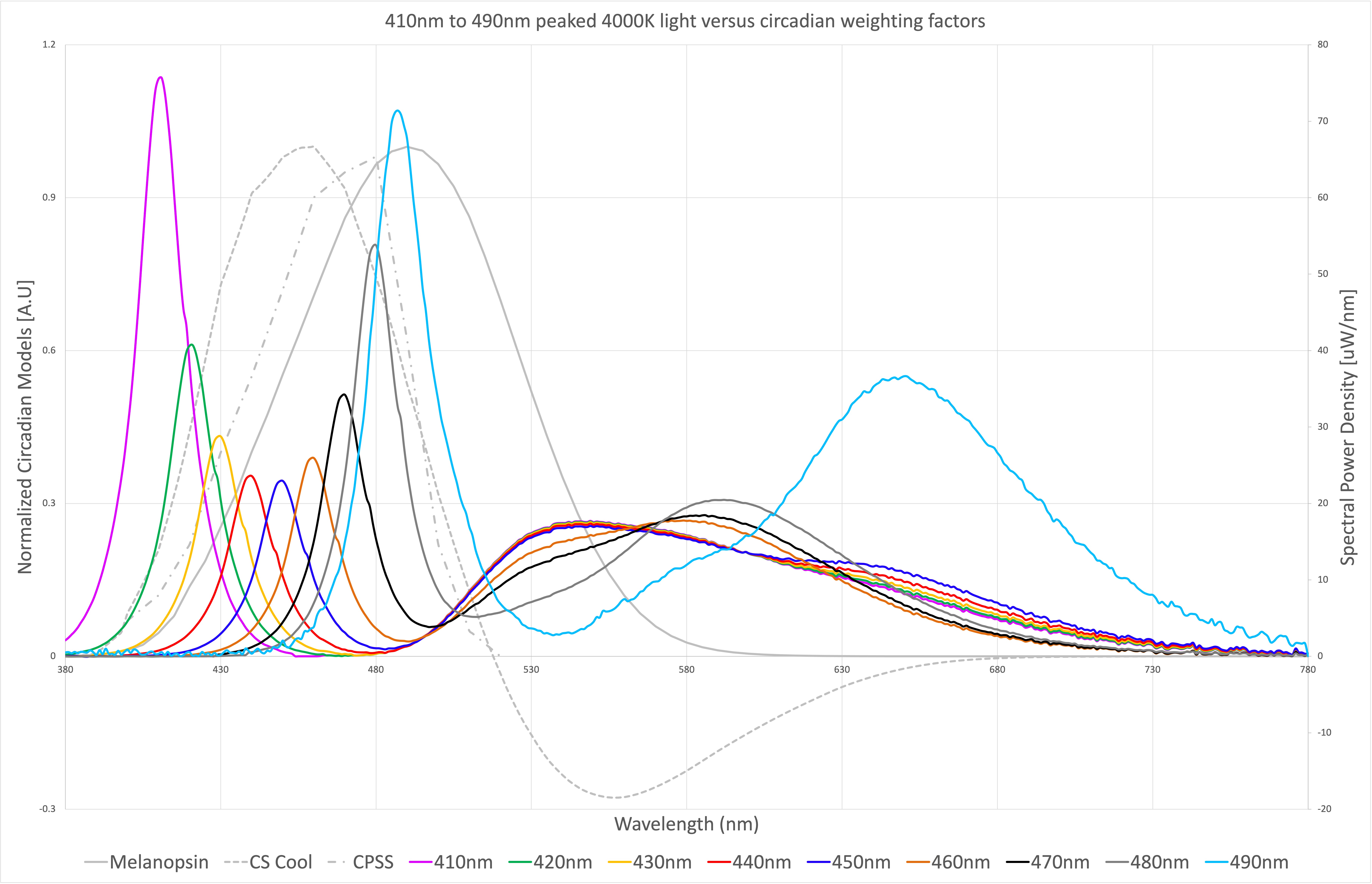

Supplement: Supplementary Figure 1 — Tristimulus sensitivity according to CIE 1931 color matching functions and melanopsin sensitivity according to CIE S 026. [file Data_Sheet_1.ZIP › Figures/Figure S11.jpg]

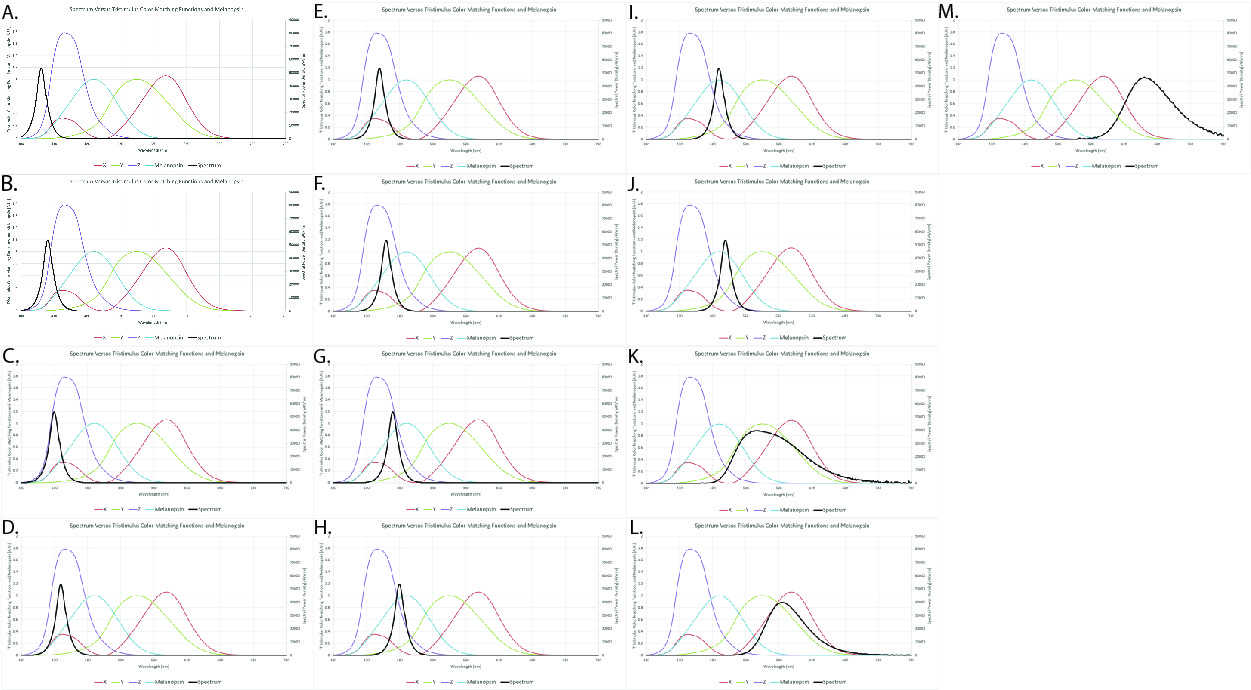

Supplement: Supplementary Figure 1 — Tristimulus sensitivity according to CIE 1931 color matching functions and melanopsin sensitivity according to CIE S 026. [file Data_Sheet_1.ZIP › Figures/Figure S3.jpg]

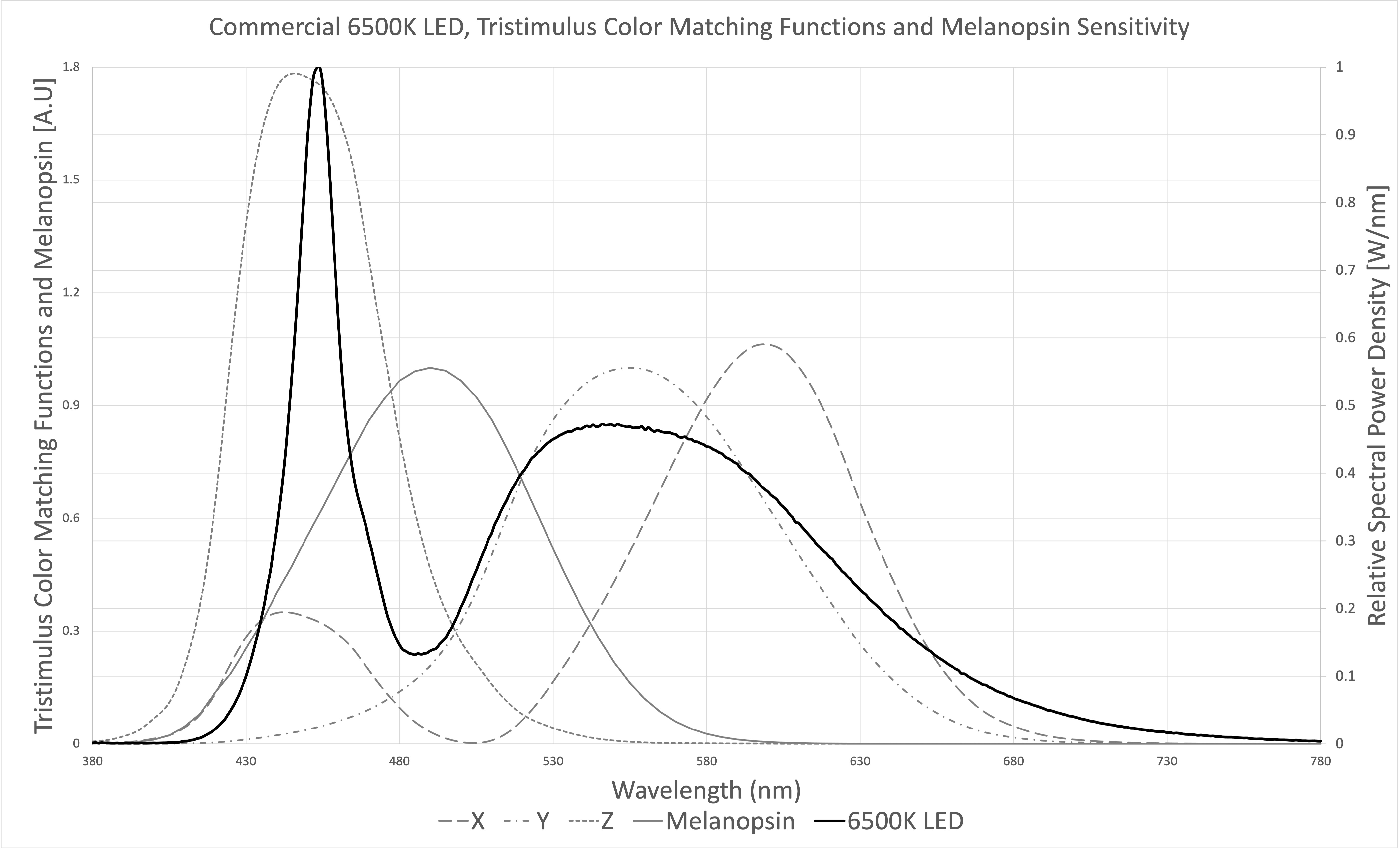

Supplement: Supplementary Figure 1 — Tristimulus sensitivity according to CIE 1931 color matching functions and melanopsin sensitivity according to CIE S 026. [file Data_Sheet_1.ZIP › Figures/Figure S2.jpg]

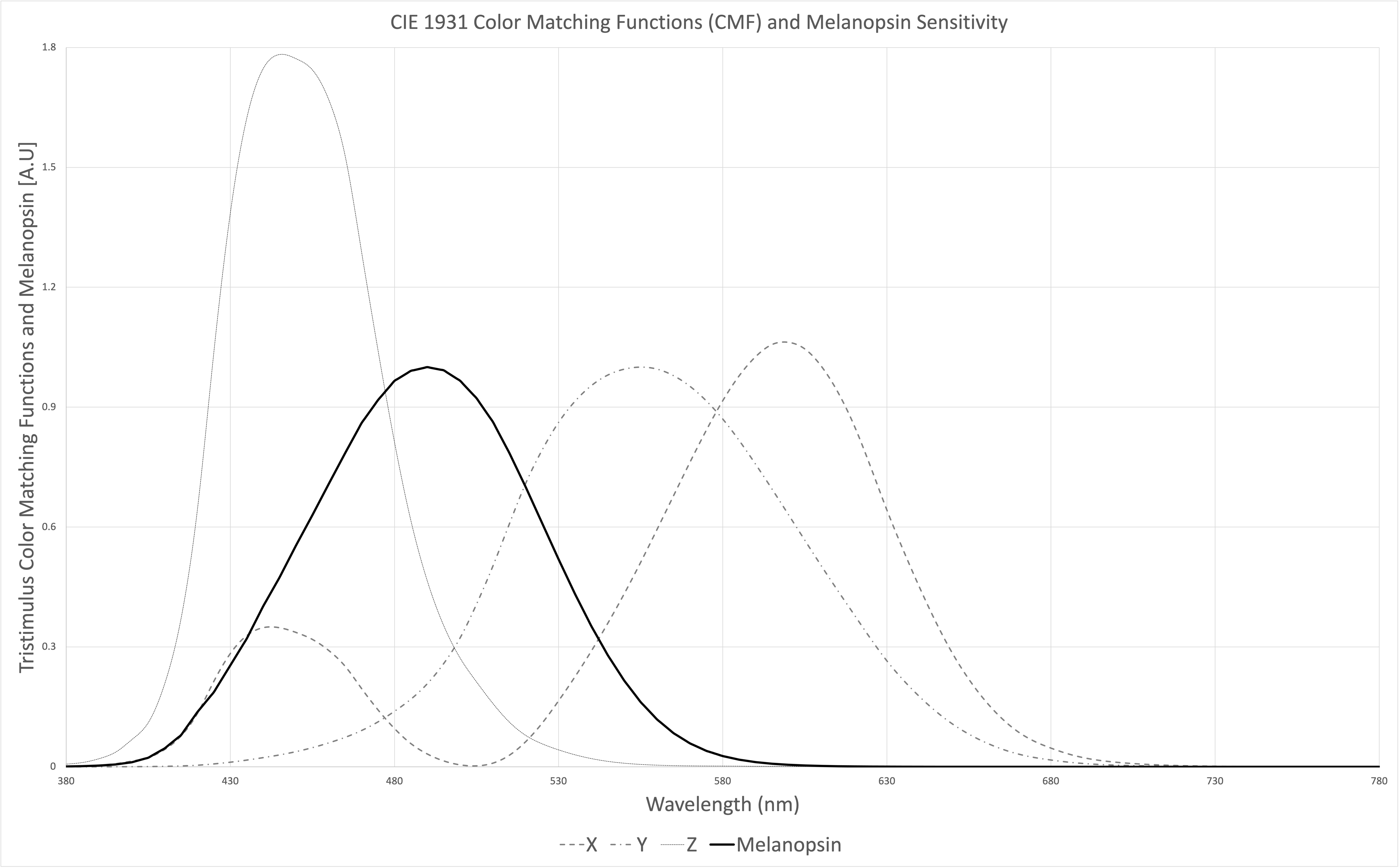

Supplement: Supplementary Figure 1 — Tristimulus sensitivity according to CIE 1931 color matching functions and melanopsin sensitivity according to CIE S 026. [file Data_Sheet_1.ZIP › Figures/Figure S1.jpg]

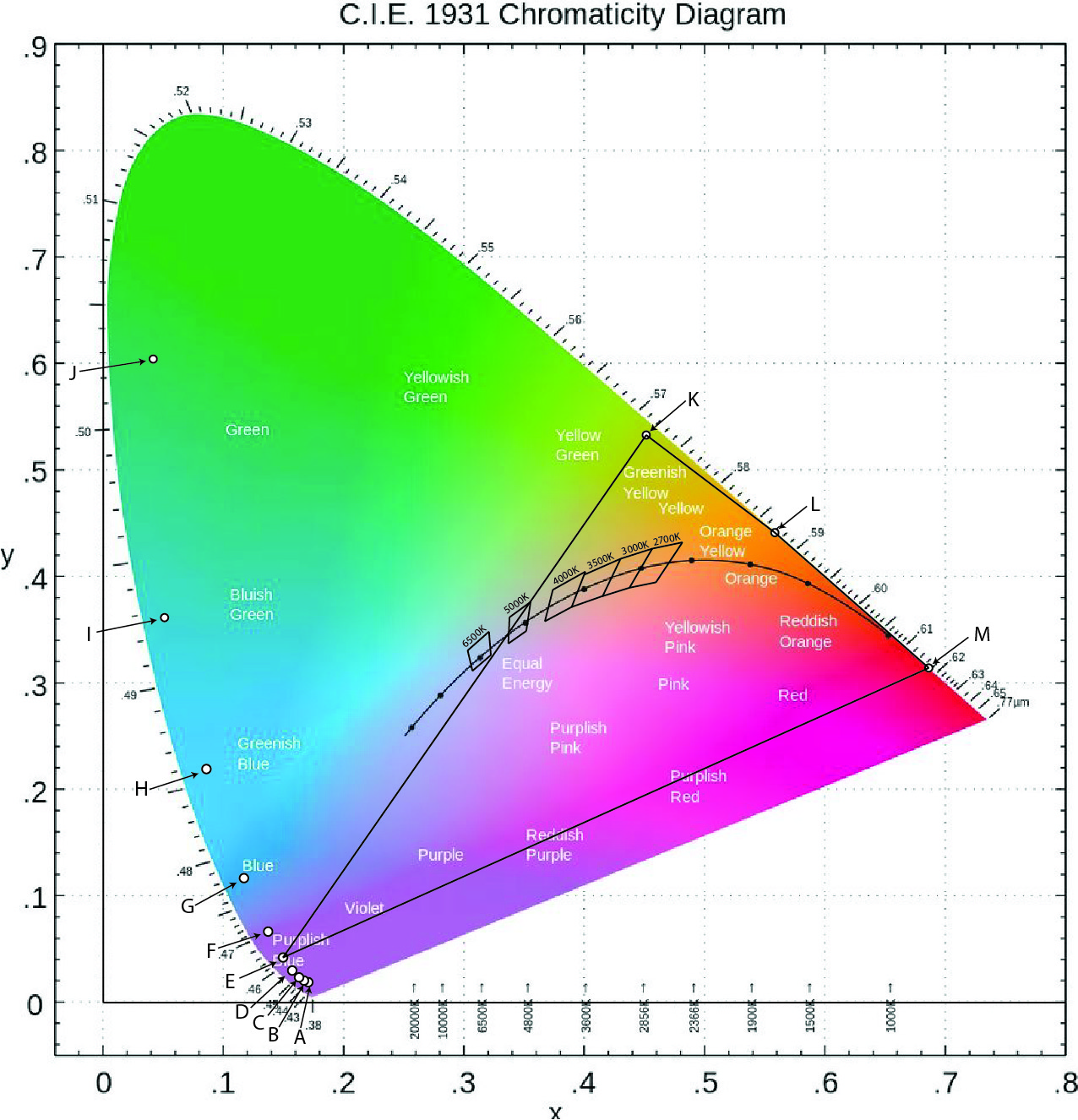

Supplement: Supplementary Figure 1 — Tristimulus sensitivity according to CIE 1931 color matching functions and melanopsin sensitivity according to CIE S 026. [file Data_Sheet_1.ZIP › Figures/Figure S5.jpg]

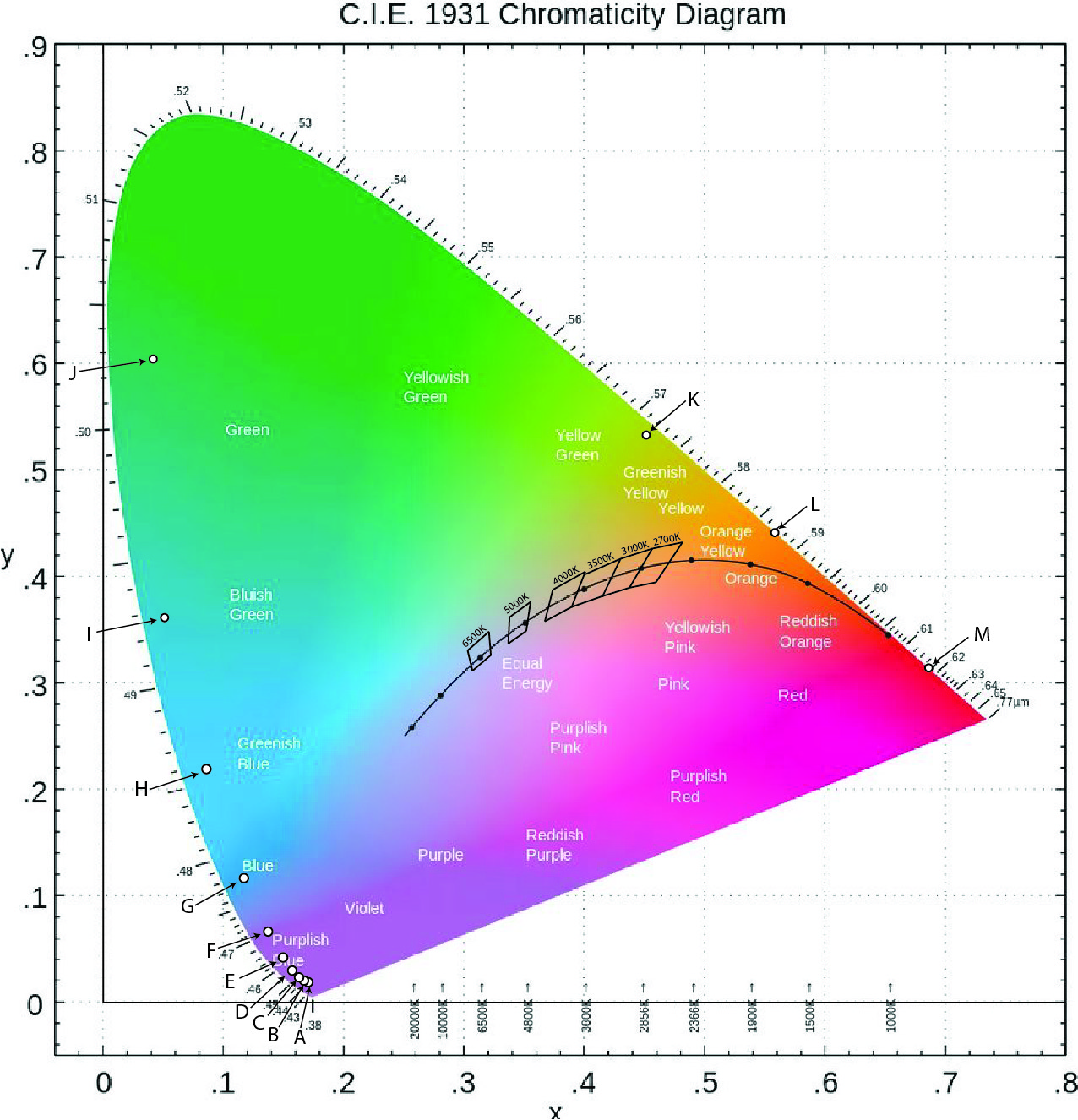

Supplement: Supplementary Figure 1 — Tristimulus sensitivity according to CIE 1931 color matching functions and melanopsin sensitivity according to CIE S 026. [file Data_Sheet_1.ZIP › Figures/Figure S4.jpg]

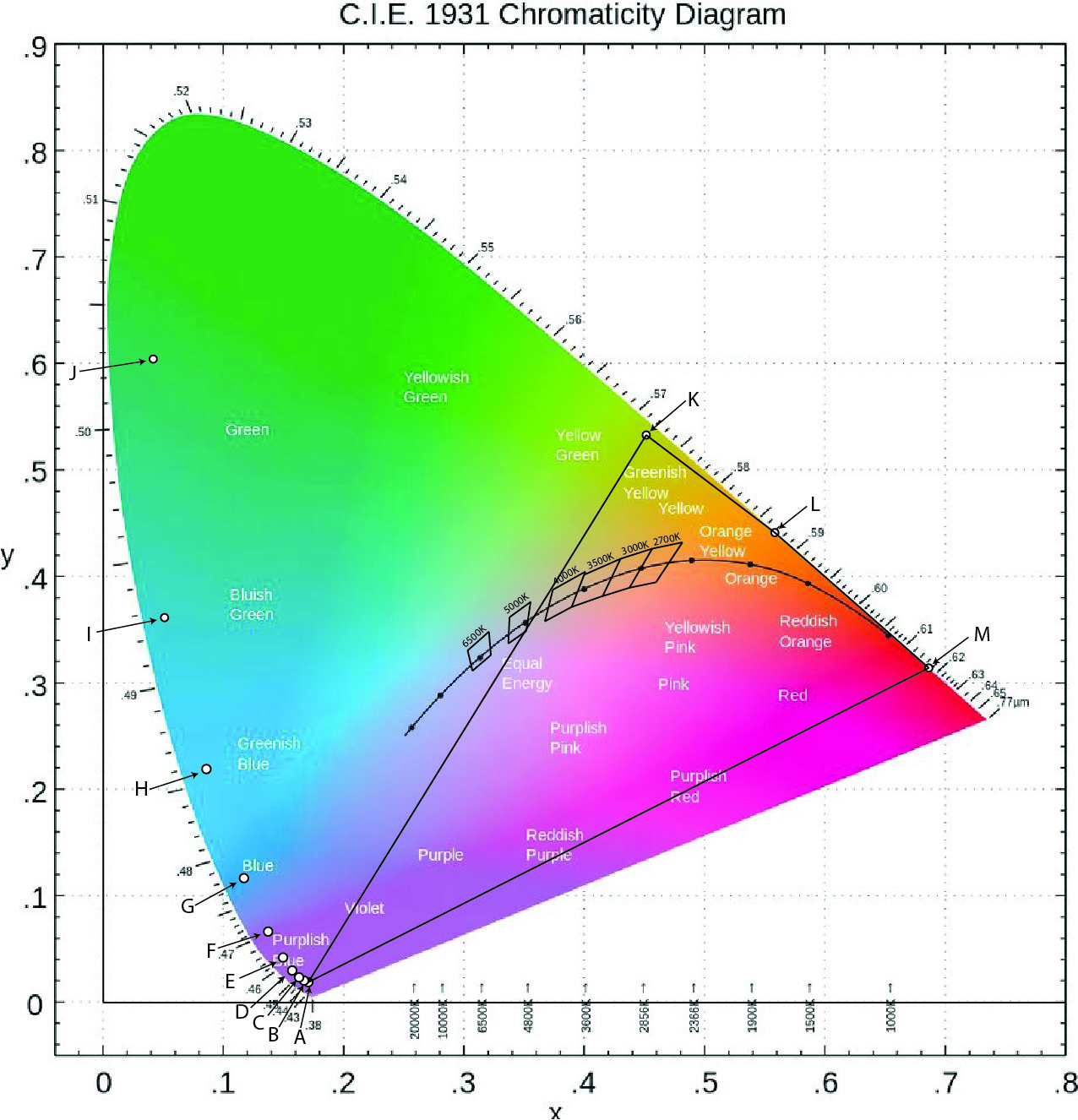

Supplement: Supplementary Figure 1 — Tristimulus sensitivity according to CIE 1931 color matching functions and melanopsin sensitivity according to CIE S 026. [file Data_Sheet_1.ZIP › Figures/Figure S6.jpg]

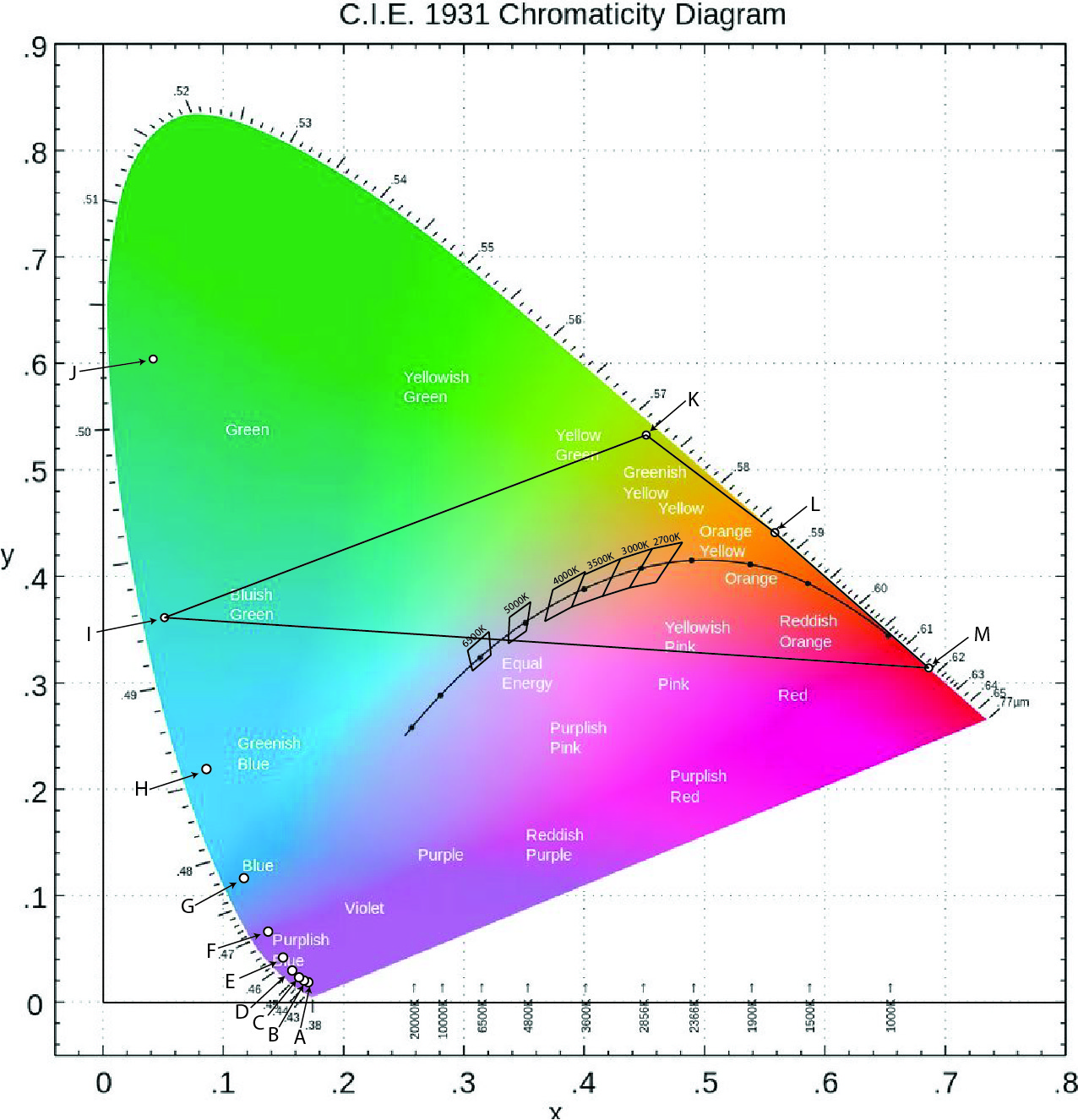

Supplement: Supplementary Figure 1 — Tristimulus sensitivity according to CIE 1931 color matching functions and melanopsin sensitivity according to CIE S 026. [file Data_Sheet_1.ZIP › Figures/Figure S7.jpg]
